# Effectiveness, Medication Patterns, and Adverse Events of Traditional Chinese Herbal Patches for Osteoarthritis: A Systematic Review

**DOI:** 10.1155/2014/343176

**Published:** 2014-01-14

**Authors:** Xuezong Wang, Songpu Wei, Ting Liu, Jian Pang, Ningyang Gao, Daofang Ding, Tieli Duan, Yuelong Cao, Yuxin Zheng, Hongsheng Zhan

**Affiliations:** ^1^Shi's Center of Orthopedics and Traumatology, Shuguang Hospital, Shanghai University of Traditional Chinese Medicine, 528 Zhangheng Road, Pudong New Area, Shanghai 201203, China; ^2^Institute of Traumatology & Orthopedics, Shanghai Academy of Traditional Chinese Medicine, Shanghai 201203, China

## Abstract

*Objective*. The aim of this study is to systematically evaluate the evidence whether traditional Chinese herbal patches (TCHPs) for osteoarthritis (OA) are effective and safe and analyze their medication patterns. *Methods*. A systematic literature search was performed using all the possible Medical Subject Headings (MeSH) and keywords from January 1979 to July 2013. Both randomized controlled trials (RCTs) and observational studies were included. Estimated effects were analyzed using mean difference (MD) or relative risk (RR) with 95% confidence intervals (CI) and meta-analysis. *Results*. 86 kinds of TCHPs were identified. RCTs and controlled clinical trials (CCTs) which were mostly of low quality favored TCHPs for local pain and dysfunction relief. TCHPs, compared with diclofenac ointment, had significant effects on global effectiveness rate (RR = 0.50; 95% CI (0.29, 0.87)). Components of formulae were mainly based on the compounds “Xiao Huo Luo Dan” (Minor collateral-freeing pill) and “Du Huo Ji Sheng Tang” (*Angelicae Pubescentis* and *Loranthi* decoction). Ten kinds of adverse events (AEs), mainly consisting of itching and/or local skin rashes, were identified after 3-4 weeks of follow-up. *Conclusions*. TCHPs have certain evidence in improving global effectiveness rate for OA; however, more rigorous studies are warranted to support their use.

## 1. Introduction

OA, which is manifested by joint pain, disability, stiffness, and/or swelling, is a common chronic disease in the elderly worldwide [1–3]. In Traditional Chinese Medicine (TCM), symptoms of OA are usually known as “*Bi*-arthralgia” or “flaccidity” [4].

Conventional therapies for the management of OA consist of exercises, weight loss, acetaminophen, and oral and topical nonsteroidal anti-inflammatory drugs (NSAIDs), as well as intra-articular injection and several other treatments [3]. Such treatments may prove ineffective in some OA patients, and NSAIDs often have gastrointestinal (GI) and cardiovascular adverse events (AEs) [3, 5], so patients are turning increasingly to complementary and alternative medicine (CAM) as treatment options for OA [5].

Some reviews provide evidence for the effectiveness of herbal medicines for OA [5–8]. At the same time, external medications for the treatment of mild or moderate OA pain have been advocated by both the Chinese Orthopedic Association (COA) and the American College of Rheumatology (ACR), because it is deemed to have relatively less AEs and is more convenient for use [3, 9]. In many topical herbal medications, the patch or plaster is most frequently prescribed [10]. TCHPs are a class of transdermal plasters that dissolve or mix different herbs with the adhesive matrix, which could then be made into a thin patch. When affixed to the injured area or acupoints, it would have a therapeutic effect locally or even systemically [4, 11].

The biological mechanisms of TCHPs for OA are known to have the following characteristics. (1) Their herbs could reach the lesions with the help of the transdermal delivery system, so they could continue to achieve an analgesic and anti-inflammatory effect [12, 13]. Some studies reveal that serum prostaglandin E2 (PGE2), interleukin-1 (IL-1), and interleukin-6 (IL-6) were decreased, while *β*-endorphin (*β*-EP) was increased in OA patients after using TCHPs [14, 15]. (2) They have a slight fixation effect and could help patients overcome fear of pain as taping [4, 16]. (3) The way of dispelling “cold evil,” removing “dampness evil,” and activating blood circulation might possibility have an impact on the immune and neurochemical systems to improve TCM syndrome [4].

At present, transdermal patches as TCHPs have been widely applied for patients with OA or chronic joint pain in China and worldwide and have accumulated abundant data in clinical practice [4, 10, 17]. To date, no comprehensive study has been documented for their effectiveness, medication patterns, and AEs, while such information would be of great value in guiding TCM practitioners or health care providers in the management of OA. Therefore, this systematic review is undertaken to investigate these important aspects of TCHPs for OA. RCTs and CCTs were chosen to evaluate the effectiveness, whereas both interventional and observational studies were included for analyzing medication patterns and AEs of TCHPs.

## 2. Methods

### 2.1. Selection Criteria

Given that chronic joint pain is the major symptom of OA and that a large number of TCHPs have listed chronic joint pain, rather than OA as their indications, it is necessary to index both OA and chronic joint pain during the search process.

Data has been pooled from the 2010 version of China Pharmacopoeia (one) and electronic databases from past decades, as they both provide clear evidence for TCHPs in the treatment of OA. When retrieving data from China Pharmacopoeia (one), TCHPs were required to show the indication of OA or chronic joint pain.

In the electronic searches, relevant articles published in English or Chinese were included if all the following criteria were met: treating OA or chronic joint pain, RCTs or observational studies; the case number enrolled into the treatment group of at least 15, and describing the main traditional Chinese herbs (commercially available or exclusively applied in the hospital). A study was excluded if, it was treating rheumatoid arthritis, gouty arthritis, or psoriatic arthritis; TCHPs were employed as one method in a combined therapy, and/or the unbalanced baseline before interventions, because it is not possible to identify the effect; it was a review or experimental articles or if there was no clinical data and/or details of herbs provided.

### 2.2. Search Strategy

The entire Academic Journals, Dissertations and Important Conference Papers Database in China National Knowledge Infrastructure (CNKI, 1989–2012), Sinomed (formerly as Chinese Biomedical Literature, CBM, 1979–2012), PubMed, and Cochrane Central Register of Controlled Trials (CENTRAL) were electronically performed up to February 2, 2012. We updated CENTRAL (Issue 7 of 12) and searched Ovid up to July 26, 2013. These databases were searched using all the possible MeSH and keywords of “osteoarthritis” and “Chinese herbal patch” (see supplementary Appendix 1 in Supplementry Material online at http://dx.doi.org/10.1155/2014/343176). Reference lists of relevant retrieved studies were extended to locate additional articles not identified in the electronic searches. Available TCHPs in the management of chronic joint pain or arthralgia in China Pharmacopoeia (one) were hand-searched.

### 2.3. Study Selection

Titles and abstracts of all records were initially checked to find relevant studies. If this information was insufficient, whole articles were retrieved to check whether the article had been missed in the initial search. Full text articles were retrieved for final analysis. The two reviewers (XZW and SPW) independently conducted study selection and assessed articles by the strategy of the established criteria.

### 2.4. Data Extraction

All articles were read and data was extracted, based on predefined standardized forms. This data mainly included first author, year of publication, title of study, simple size, types of trial, treatment and control group, methodological quality, eligibility criteria, outcome measures, name and components of TCHPs, descriptions of effectiveness, details of AEs, and follow-up period for each study.

A classical textbook was referred to, to standardize the herbal name involved in all TCHPs [18]. Synonyms of herbs were merged and different herbs were distinguished. Matrices such as honey, rosin, and licorice were excluded, because they act as processing materials, usually with no detailed dosage available.

### 2.5. Quality Assurance

All authors worked together to develop relevant MeSH, keywords of each database, and screening methods of citations. All works were applied independently by two authors to screen the full texts of articles. In case of disagreement, the two reviewers tried to discuss and achieve a consensus. When a consensus could not be reached, a third reviewer (YLC) was consulted to make the final decision.

### 2.6. Analysis Plan

According to the unique philosophical and methodological characteristics of TCM [19], evidence of effectiveness, medication patterns, and related AEs of TCHPs have been synthesized, respectively.

The qualities of the reports of RCTs and CCTs were assessed by the Cochrane Collaboration's tool for assessing risk of bias to address the following domains: random sequence generation, allocation concealment, blinding, incomplete outcome data, selective reporting, and other bias. Judgments were categorized as low risk of bias, high risk of bias, or unclear risk of bias. If insufficient information was prevented to make judgment, trials were categorized into high risk of bias; if adequate reporting was provided, trials were categorized to low risk of bias, and the rest were recorded unclear risk of bias.

The effectiveness of TCHPs for OA was defined as a significant improvement compared to the placebo, NSAIDs, or other therapeutic interventions (e.g., infrared therapy) in outcomes of OA pain, dysfunction, or global effectiveness rate (TCM syndrome). Statistically, the difference between the intervention group and the control group was considered to be an improvement (*P* < 0.05 or *P* < 0.01); noninferiority results of TCHPs group compared to NSAIDs were also included.

Data was pooled using MD with 95% CI for continuous outcomes or RR with 95% CI for binary outcomes through Revman 5.2 software. Meta-analysis would be done if RCTs had a good homogeneity and the funnel plot would explore publication bias if enough trials were identified. When the *I* ^2^ was greater than 50%, higher levels of statistical heterogeneity were existed and random effects model was used. When *I* ^2^ was less than 50%, a fixed effects model would be more appropriate. RCTs and observational studies were included for analyzing the medication patterns and AEs in all included TCHPs.

## 3. Results 

### 3.1. Description of Included TCHPs

**Figure 1:**
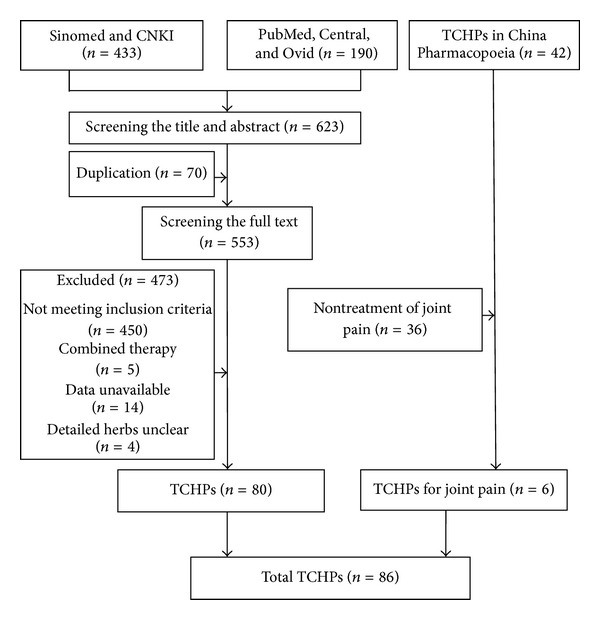
Flow diagram of search of TCHPs for OA.

623 citations were initially screened (433 in Sinomed and CNKI and 190 in PubMed and CENTRAL). Among them, 70 citations were duplicated and 473 citations were excluded, mainly due to not meeting inclusion criteria. The 2010 version of China Pharmacopoeia (one) recorded 42 kinds of topical TCHPs, but only 6 documented the indication of chronic joint pain. Hence, a final library of 80 articles from electronic database and 6 records from China Pharmacopoeia (one) remained for evidence synthesis (supplementary Appendix 2). In other words, 86 kinds of TCHPs were involved in our final analysis (Figure 1). Of the 86 TCHPs, 22 were commercially available, whereas the remainders were exclusively applied in the hospital. Six kinds of TCHPs were recorded in China Pharmacopoeia (one), with the indication of chronic joint pain rather than OA name. On the contrary, varieties of new TCHPs have been reported in the treatment of OA in our literature search but were not recorded by China Pharmacopoeia (one).

### 3.2. Description of Included RCTs and CCTs

80 articles included 44 RCTs, 35 observational studies, and 1 study protocol for an RCT [10]. The number enrolled into TCHPs group was 9723 patients.

#### 3.2.1. Description of Included RCTs

36 studies declared a greater effect compared with the control group, of which 5 studies used diclofenac ointment [20–24] and 7 studies reached noninferiority effect [25–31], including 3 studies using diclofenac ointment and 1 using diclofenac [25–28]. The characteristics of TCHPs compared with diclofenac ointment or placebo were listed in Table 1 and 686 participants were involved. The duration of treatment ranged from 7 to 42 days [4, 28]. 18 studies provided information on patients' syndrome differentiations (TCM-Zheng) as the basis of effectiveness for using TCHPs [4, 12, 21, 22, 25–27, 29, 32–41]. For example, when applied for knee OA with syndrome of kidney deficiency and blood stasis, the “Huo Xue Hua Yu” patch (Gao) significantly improved total scores of TCM syndrome and OA symptoms compared with diclofenac ointment [21].

#### 3.2.2. Description of Included CCTs

According to the study design checklist and guidance about collecting the information of the studies (Chapters 13.2 and 13.4), apart from 23 case series, 10 nonrandomized controlled trials (NRCT) [33, 34, 42–49] and 2 interrupted-time-series (ITS) studies [12, 50] were identified among 35 observational studies. A summary of the included CCTs were listed in Table 2. In total, 1607 participants were included. The duration of treatment ranged from 9 days to 5 weeks, even with 1-year follow-ups [47]. A three-arm study concerning knee pain and range of motion (ROM) about “Gu Ci” patch versus control patch was obtained [43]. One reported using “Gu Bi” patch for the chronic joint pain of knee, ankle, and shoulder [47]. In these studies, the outcome measure was unclear and stated rare in 4 trials [42, 43, 48, 49].

### 3.3. Methodological Assessments

#### 3.3.1. Assessments of Risk of Bias of Included RCTs

**Figure 2:**
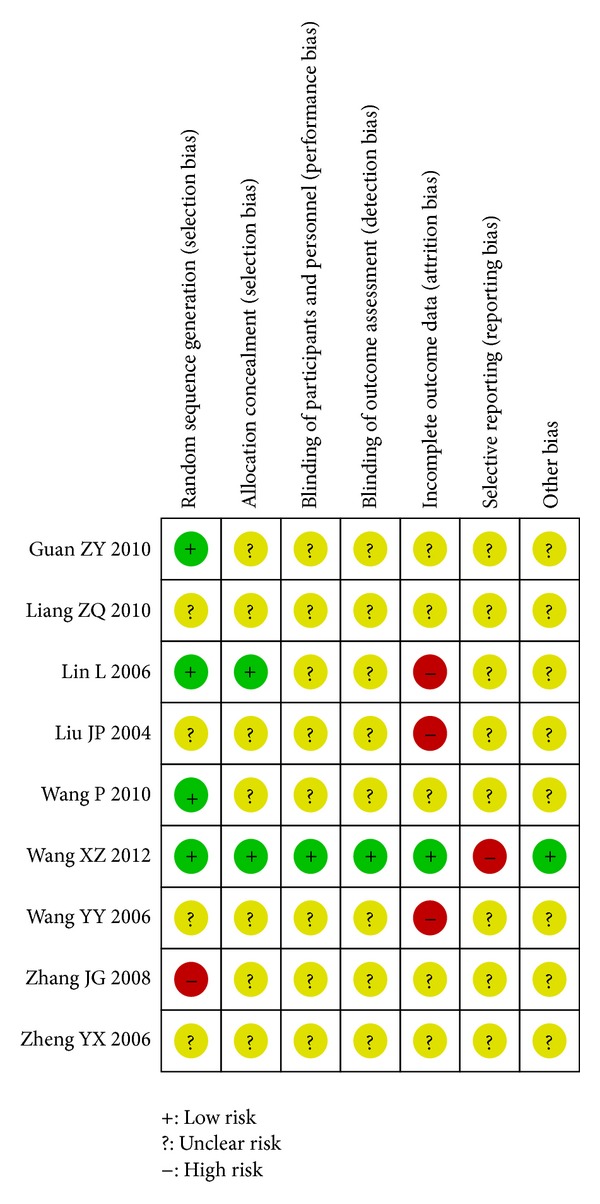
Risk of bias summary of TCHPs versus diclofenac ointment or placebo.

RCTs were generally of poor methodological quality or were poorly reported (supplementary Appendix 3). The randomized allocation of participants was declared in all included RCTs. However, only 12 trials mentioned methods for sequence generation, such as random number table [21, 22, 30, 35, 36, 41, 51, 52] or computer software [4, 10, 23, 25]. Three trials were of single-blind design [40, 51, 53] and 2 were of double [4, 31]. Two trials were assessed as having concealment and obtained research ethics approval [4, 36]. Nearly, all the trials provided baseline data for the comparability among groups. Four trials reported information on withdrawal/dropout [20, 25, 28, 35]. Majority of studies lacked the information for dropouts and outcome measures were quite varied. Most studies have conducted follow-ups of 3 to 4 weeks. Risk of bias summary of TCHPs versus diclofenac ointment or placebo was shown in Figure 2.

#### 3.3.2. Quality Assessments of CCTs

Ten NRCT [33, 34, 42–49] and 2 ITS studies [12, 50] were assessed (supplementary Appendix 3). As reported, 1 mentioned randomization; actually it was an NRCT mainly for within group comparison [12]. Only 6 reported comparability at baseline [12, 33, 34, 44, 46, 49]. Biases were found at statistical methods [46], unreasonable formulations compared with TCHPs, such as using tablet [43], pill [44], and sodium hyaluronate [42]. Four used eligibility criteria with diagnosis/grade of OA and TCM syndrome differentiations [12, 33, 34, 50]. No studies reported information on withdrawal/dropout and described precisely how confounding factors were measured or fitted as covariates to control.

### 3.4. Effect Estimates of RCTs

All the RCTs demonstrated a positive effect, favoring TCHPs for OA, except one study protocol [10]. Pain relief was the most frequently reported positive benefit of TCHPs [21, 24, 31, 35, 36, 38, 40, 41, 53–56]. The onset time of reducing pain was from 4.02 hours to 15.40 hours [56, 57], and the effect could be maintained from 2.30 days to 6.77 days [53]. The reported outcome measures included local pain relief, dysfunction relief, and overall effectiveness rate. According to the analysis plan and established outcome measures, firstly, TCHPs were compared with diclofenac ointment or placebo. Effect estimates of TCHPs compared with diclofenac ointment or placebo for OA were shown in Table 3.

For the global effectiveness rate, as most studies have used diclofenac ointment as the control group, the meta-analysis has been applied. Concerning specific outcomes of local pain or dysfunction relief, there was high heterogeneity in the aspects of control group and methodology design, so data was only synthesized using MD or RR with 95% CI rather than the meta-approach.

#### 3.4.1. Local Pain Relief

Four trials with 5 comparisons including 342 participants reported the favorable effect of TCHPs individually versus diclofenac ointment or placebo [4, 8, 21, 23]. Among them, local pain was reduced by Lequesne's Index, clinical research guidelines of traditional Chinese patent drug by the 21st day [RR 0.50, 95% CI 0.10 to 2.56; *n* = 91] [23], and the Hospital for Special Surgery (HSS) by the 14th day [MD 0.00, 95% CI −1.09 to 1.09; *n* = 60] [21]. One study demonstrated a similar effect by the Western Ontario and McMaster Universities Arthritis Index (WOMAC), compared with diclofenac by the 42nd day [MD −1.14, 95% CI −3.56 to 1.28; *n* = 60] [28]; however, 1 three-arm study showed no significant improvement in the aspect of pain compared with placebo in the visit of the 6th day [MD −1.44, 95% CI −1.69 to −1.19; MD 1.08, 95% CI 0.83 to 1.33; 1 trial; *n* = 150] [4].

#### 3.4.2. Local Dysfunction Relief

Three studies with 7 comparisons involving 270 participants have identified TCHPs compared with diclofenac ointment [8, 21] or placebo [4] in the aspect of improving dysfunction of knee OA. The “Huo Xue Hua Yu” patch was documented as giving an improved effect on ROM and flexion deformity over a 14-day treatment as compared to diclofenac ointment [MD 0.06, 95% CI −0.29 to 0.41; MD 0.06, 95% CI −0.40 to 0.55; 1 trial; *n* = 60] [21]. One demonstrated similar effect compared with diclofenac ointment for the treatment over 42 days [MD −1.30, 95% CI −6.46 to 3.86; *n* = 60] [28], but one showed no significant improvement in the aspect of stiffness and physical function compared with placebo in the visit of the 6th day [4].

#### 3.4.3. Meta-Analysis for Global Effectiveness Rate

**Figure 3:**
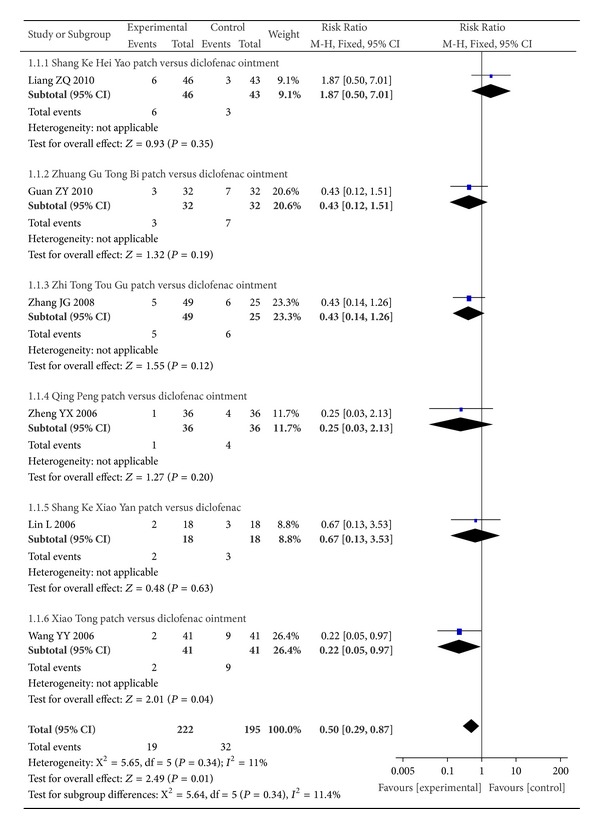
Forest plot of comparison of TCHPs versus diclofenac ointment for OA in global effectiveness rate.

**Figure 4:**
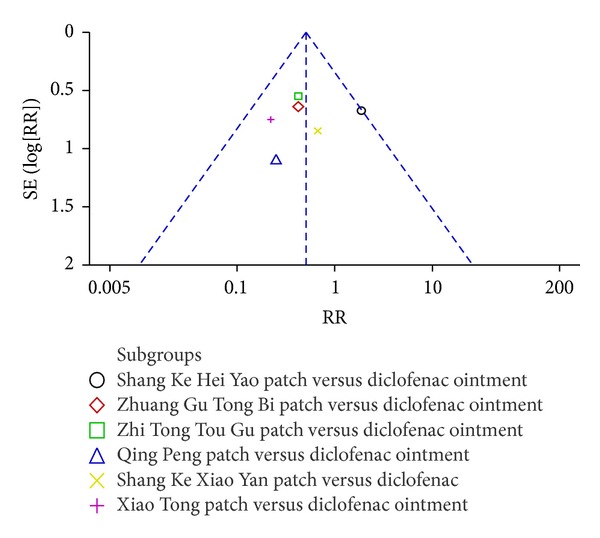
Funnel plot of comparison of TCHPs versus diclofenac ointment for OA in global effectiveness rate.

Six trials with 6 comparisons including 417 participants demonstrated the effectiveness rate of TCHPs versus diclofenac ointment [20, 22, 23, 25, 26, 58] (Figure 3). As reported, 4 trials showed that TCHPs had better effect on the global effectiveness rate [20, 22, 23, 58]. Two trials demonstrated noninferiority results favoring TCHPs [25, 26]. As *I* ^2^ was less than 50%, lower levels of statistical heterogeneity were denoted and the fixed effects model was used for meta-analysis. Collectively, the results showed that TCHPs had significant effects on the global effectiveness rate [RR = 0.50, 95% CI 0.29 to 0.87; 6 trials; *n* = 417]. However, the funnel plot of comparison of TCHPs versus diclofenac ointment for OA demonstrated asymmetry, suggesting the possibility of publication bias (Figure 4).

Compared to other RCTs interventions, TCHPs could reduce local pain by the 10th day [36], local pain, joint swelling, and locomotor disability by the 14th day [31], local pain by the 21st day [55], locomotor disability by the 14th/28th day [27, 41], local pain, stiffness, and dysfunction by the 28th day [35], local pain, locomotor disability, and burning sensation [24], local pain by the 60th day. They could also improve walking distance and ROM by the 60th day [54].

### 3.5. Effect Estimates of TCHPs for OA about CCTs

Results of favored TCHPs for OA about CCTs were shown in Table 4. Global effectiveness rate from 75% to 97.91% was the main outcome [46, 50]. Kuang studied 48 cases of “Zhong Tong Xiao Babu” patch compared with 46 cases of its old dosage form in the treatment of knee OA; it had a better global effectiveness rate of TCM than the old one [RR 0.43, 95% CI 0.14 to 1.29] at 10 days and could improve pain [MD −0.96, 95% CI −1.65 to −0.27], [MD −0.90, 95% CI −1.56 to −0.24] and function [MD −0.90, 95% CI −1.18 to −0.62], [MD −1.00, 95% CI −1.27 to −0.73] at 5 and 10 days, but no statistically significant difference of the swelling were showed [MD −0.10, 95% CI −0.32 to 0.12; MD −0.01, 95% CI −0.21 to 0.19] [12], respectively. Compared with “She Xiang Zhuang Gu” patch on the basis of visual analog scale, “Gu Ci” bapu patch also showed an improvement of global effectiveness rate in the treatment of knee OA [RR 0.67, 95% CI 0.26 to 1.68; *n* = 72] and [RR 0.57, 95% CI 0.18 to 1.78; *n* = 72] and could improve pain [MD 0.21, 95% CI −0.02 to 0.44], [MD −1.20, 95% CI −1.34 to −1.06] at 5 and 10 days, respectively [50]. One patch showed significant difference compared with sodium hyaluronate 20 mg per week with a course of treatment of 5 weeks [RR 0.48, 95% CI 0.26 to 0.89; *n* = 176] [42]. The intensity of pain of knee about “Gu Ci” patch compared with control patch was obtained at a three-arm study [RR 0.48, 95% CI 0.26 to 0.89; *n* = 69] and also showed significant difference on ROM [RR 0.46, 95% CI 0.19 to 1.08; *n* = 69] [43]. “Xi Tong Kang” patch compared with “Tong Luo Qu Tong” patch on global effectiveness rate was [RR 0.47, 95% CI 0.18 to 1.27; *n* = 108] at 28 days [34]; similarly, there were statistically significant differences in 7 trials with the course of treatment from 14 days to 5 weeks [33, 44–49].

### 3.6. Medication Patterns

Based on TCM clinical pathways and the textbook [17, 59], there are mainly two types of therapeutic principles for the treatment of OA.

One was a class of dispelling cold-damp, promoting blood circulation and strengthening analgesic efficacy to treat syndrome of cold-damp stasis blockage (wind-cold-damp *Bi*-arthralgia or tendons-muscular stasis) with local joint pain, swelling or effusion, feeling of heaviness, and functional impairment. All these symptoms are most likely to become worse on cloudy and rainy days, as well as a preference for warmth and pressing, unchangeable skin color, thick tongue, thin or greasy tongue coating, thin or string pulse condition, and so forth. Such targeted TCHPs were the “Gou Pi” patch, the “Fu Fang Nan Xing Zhi Tong” patch, and the “Hei Yao” patch [4, 26, 60].

Another small portion was a class for clearing heat and damp, cooling blood, and relieving pain to treat syndrome of wind-damp-heat *Bi*-arthralgia with pain or tingling, increased skin temperature, effusion or swelling, functional impairment, associated with local burning, thirst or lack of thirst, bitter mouth, dry stool and yellow urine, red tongue, thin yellow or yellow greasy tongue coating, slippery-quick or string pulse condition, and so forth. Targeted TCHPs were the “San Huang” patch, the “Huang Bo Wu Wei” patch, and the “Xi Tong Ning” patch [24, 44, 61].

All TCHPs included in the survey involved 179 herbs with 981 frequencies. On average, 12 kinds of herbs were included (ranging from 2 to 31) [62, 63] and every patch contained 7 g–15 g of herbs [64, 65], but no detailed dosage of each herb was available. Frequency of each herb was added and the top 20 were listed based on the accumulated frequency (Table 5). Among them, the top 7 above 30 percent were “Chuan Wu” (*Radix Aconiti*), “Cao Wu” (*Radix Aconiti Kusnezoffii*),“Mo Yao” (*Myrrha*), “Ru Xiang” (*Olibanum*), “Dang Gui” (*Radix Angelicae*), “Bing Pian” (*Borneolum Syntheticum*), and “Chuan Xiong” (*Rhizoma Ligusticum chuan xiong*).

It is clear that the top 7 herbs are suited for the syndrome of cold-damp stasis blockage: Chuan Wu and Cao Wu are used to dispel the evil of wind, damp, and cold as well as relieve pain; Mo Yao and Ru Xiang promote blood circulation to achieve analgesic effect; Chuan Xiong accelerates blood circulation and qi and relieves pain; the effects of Dang Gui enrich the blood and promote blood circulation; and the emitting of Bing Pian stimulates drug absorption, so they have the effect of promoting blood circulation and dredging meridians, eliminating swelling and pain with the help of a transdermal delivery system [12, 13, 50].

After categorizing the different herbs according to their actions, the most used ones are those which have the effect of dispelling cold-damp and promoting blood circulation [18] (Table 6). If we investigated the formula, it is clear that they are mainly based on “Xiao Huo Luo Dan” (minor collateral-freeing pill) and “Du Huo Ji Sheng Tang” (*Angelicae Pubescentis* and *Loranthi *decoction).

### 3.7. Adverse Events

For AEs of patches in 80 literature sources, 38.75% studies (31 of 80) did not mention whether they had monitored AEs or not, 32.50% studies described AEs, whereas the remaining 28.25% reported no incidence of AEs. Among all the reports, detailed information of AEs was identified (Tables 1 and 7). Apart from a special therapy of blistering [41], the incidence of AEs ranged from 0.66% to 12.24% [20, 36]. When we compared the incidence of withdrawal and AEs of TCHPs with diclofenac ointment or placebo, it was found that the TCHPs group was more than the placebo [7.56% (9/119) versus 0.00% (0/30)] [4] and less than diclofenac ointment [2.82% (2/71) versus 8.99% (8/89)] [25, 28, 58].

**Figure 5:**
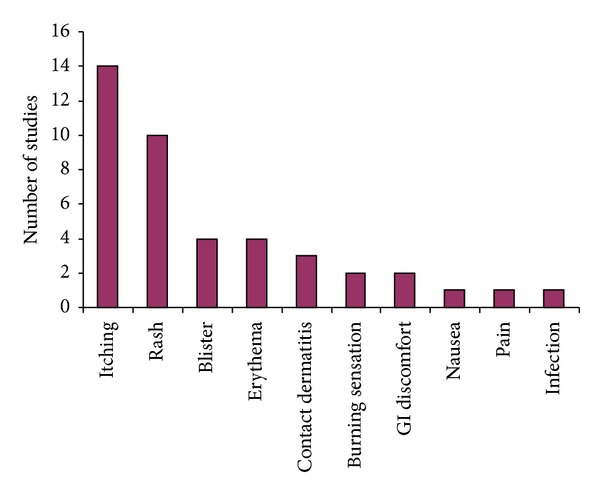
Number of studies recording different AEs for TCHPs in the treatment of OA.

Ten kinds of AEs were identified in 49 articles (Figure 5). The most common were local itching in 28.57% (14 of 49) articles and rashes or papules (20.41%). The following were blister (8.41%) [66–69], erythema (6.12%) [4, 20, 53], contact dermatitis (6.12%) [4, 64, 70], burning sensation (4.08%) [71, 72], GI discomfort (4.08%) [73, 74], nausea (2.04%) [74], and/or pain (2.04%) [41], respectively. Infection was reported for redness, oozing, and purulent [49]. Withdrawal or drop-out occurred for blister and contact dermatitis [4, 28, 35, 64, 66, 70] and even for unsatisfied efficacy [28, 73].

## 4. Discussion

This is the first study to systematically investigate the evidence of effectiveness and AEs and analyze medication patterns of TCHPs for OA. As there is no current data to support a particular group of patches possessing overwhelming efficacy in the treatment of OA, and since there was no meta-analysis available, we therefore comprehensively sourced all the evidence from both clinical studies and China Pharmacopoeia (one).

The review showed that TCHPs, which were mostly of low quality, could obviously improve global effectiveness rate, reduce local pain, and/or raise function comparing with diclofenac ointment or placebo. The result of meta-analysis showed a statistically significant improvement to global effectiveness rate of OA participants [RR = 0.50, 95% CI 0.29 to 0.87; 6 trials; *n* = 417]. Formulae of TCHPs were mainly based on Xiao Huo Luo Dan and Du Huo Ji Sheng Tang. The incidence of AEs was less than diclofenac ointment group. Ten AEs mainly concerning itching and/or rashes of local skin were identified.

The efficacy of Chinese herbal medicine for OA was found to be better than or similar to conventional therapies [8, 75]. Consistent with our study, a previous review which detected the external use of Chinese herbal medicine has also documented a good efficacy and safety for OA. Apart from TCHPs, it has included other intervention methods and the control group was also of diversity (NSAIDs, Cox-2 inhibitors, sodium hyaluronate intra-articular injection, and pain spot blocking), so results were combined and the incidence of AEs was therefore smaller than that of our result (1.87% versus 2.82%) [8].

Although the review demonstrates that TCHPs could ease OA symptoms, it may be affected by low methodological quality of included RCTs and potential publication bias indicated by asymmetry funnel plot. It is known that low methodological studies indicated greater differences between test and control group than those well conducted [76]. Therefore, further trials with more rigorous design and unpublished studies are needed in this area.

This study has documented those herbs with the effect of dispelling cold-damp, promoting blood circulation, and relieving pain, such as Chuan Wu, Cao Wu, Mo Yao, Ru Xiang, and Dang Gui which were the major components of TCHPs. Furthermore, results of analyzing both the herbs' frequencies and their effects were consistent. Formulae of TCHPs were mainly based on Xiao Huo Luo Dan and Du Huo Ji Sheng Tang. Xiao Huo Luo Dan was documented in formulae by the Taiping Pharmaceutical Bureau for Benevolence to relive pain, so that the wind-cold-damp evil might be relieved [77]. Pharmacological studies have confirmed its anti-inflammatory, analgesic, and immunosuppressive role [78], so it has a good therapeutic effect on the early and mid-OA [79]. Du Huo Ji Sheng Tang is derived from “Bei Ji Qian Jin Yao Fang.” Topical use is mainly for removing wind-damp evil to warm and dredge meridians and cure *Bi*-arthralgia [4, 80]. Whether via oral administration or topical use, its efficacy in the treatment of knee OA has been confirmed [81–83].

It is commonly believed that the AEs of TCHPs should be less and TCHPs is convenient for topical use, but there are still 10 kinds of self-reported AEs identified by this study. On the one hand, a large part of these studies have no description of AEs, indicating insufficient information about monitoring and reporting of AEs, and, on the other hand, over half of these studies (53.06%; 26 of 49) have demonstrated AEs. Given that OA is a chronic progressive disease, results from current relatively short term studies (mostly 3-4 weeks) seem to have underestimated the incidence of AEs. Nevertheless, for the incidence of AEs, the TCHPs group was less than diclofenac ointment (2.82% versus 8.99%) [25, 28, 58]. AEs reported were mild to modest, as the majority of allergic reactions were local skin itching and/or rashes.

TCHPs are warm and dry in nature and drastic in potency, so it is appropriate for patients with a strong constitution and should be applied with caution for those with heat syndrome due to damp obstruction, Yin deficiency and/or for pregnant women. As Chuan Wu, Cao Wu, or Ma Qian Zi has potential kidney or liver toxicity, it should be used with caution when administered in high-dose or for long-term use [77]. If blister, itching, and/or rash occurs in the local skin, it should be stopped immediately.

In the included articles, all the prescriptions of TCHPs were based on the medication patterns, and only 18 studies provided information on patients' syndrome differentiation as the basis of effectiveness for using TCHPs [4, 12, 21, 22, 25–27, 29, 32–41]. It is recommended to use TCHPs based on both OA symptoms and TCM syndrome if applicable. Otherwise, the effectiveness might be decreased and the incidence of AEs might be increased theoretically, although there are currently no reports concerning this issue.

This study has had several limitations. Firstly, diagnosis/grade of OA was not clear in most of the trials for insufficient reporting of either ACR or COA criteria and Kellgren-Lawrence scale, so findings would limit the generalization to OA population. It is noteworthy that OA is a chronic longer-term condition; it therefore remains presenting symptoms and signs of disease-stage with the majority of OA participants. Secondly, no data was available to support which Chinese herbs were superior to others. Hence, the top 7 most common herbs and components of formulae were listed so as to provide a broader reference to future study. Thirdly, the funnel plot showed asymmetry, which indicated the possibility of publication bias existed in this review. Although this might also be induced by language bias as we only retrieved literatures in Chinese and English, factors from relatively small sample sizes of these studies could not be excluded. Finally, the included RCTs were low in quality and a meta-analysis could be only applied in the aspect of global effectiveness rate rather than in specific outcome measures such as pain and dysfunction. However these have been synthesized through standard RR or MD. Moreover, the initial evidence for the effectiveness and clue of AEs as well as specific components of formulae may provide beneficial references to practitioners when using TCHPs.

## 5. Conclusions 

In summary, this review suggests that TCHPs have certain evidence for OA in improving global effectiveness rate. Components of formulae were mostly based on Xiao Huo Luo Dan and Du Huo Ji Sheng Tang. The main AEs were itching and/or rashes of local skin, but further studies concerning AEs, effectiveness, and medication patterns are warranted to support their use.

## Supplementary Material

Search process and risk of bias assessment of TCHPs.

## Figures and Tables

**Table 1:** Characteristics of TCHPs versus diclofenac ointment or placebo for OA in included RCTs.

First author (year)	No. (M/F)	Age (yrs)	Disease duration	Comparisons	Outcome measures	AEs
Guan, 2010 [22]	*T*: 9/24; *C*: 6/23	*T*: 53.15 ± 12.76; *C*: 54.28 ± 11.12	*T*: 18.3 ± 9.36; *C*: 17.5 ± 10.22	Zhuang Gu Tong Bi patch versus diclofenac ointment for the treatment of 28 days	Global effectiveness rate	No AEs were identified

Liu, 2004 [28]	*T*: 12/18; *C*: 10/20	*T*: 46.8; *C*: 48.7	*T*: 8.2; *C*: 9.2	Self-prescribed herbal patch versus diclofenac ointment for the treatment of 42 days	Global effectiveness rate and function	One case exited because of lack of effect in treatment group (1/30; 3.33%); three cases of skin allergic reactions exited in control group (3/30; 10.00%)

Lin, 2006 [25]	*T*: 7/11; *C*: 5/13	*T*: 42~85; *C*: 46~81	Not reported	Shang Ke Xiao Yan patch versus diclofenac for the treatment of 28 days	Global effectiveness rate	Two patients exited in the medium term of treatment in diclofenac group due to AEs (2/18, 11.11%)

Long, 2006 [26]	*T*: 15/31; *C*: 16/27	42~67 (mean = 57.7) (for all)	Not reported.	Shang Ke Hei Yao patch versus diclofenac ointment for the treatment of 28 days	Global effectiveness rate	No AEs were identified

Wang, 2010 [21]	*T*: 4/26; *C*: 5/25	*T*: 57.20 ± 8.10; *C*: 58.60 ± 8.00	5 d~3 yrs (for all)	Huo Xue Hua Yu patch versus diclofenac ointment for the treatment of 14 days	Pain, range of motion (ROM), and flexion deformity	Not reported

Wang, 2012 [4]	*T*1: 7/53; *T*2: 4/56; *C*: 3/27	*T*1:58.5 ± 7.7; *T*2: 59.6 ± 6.1; *C*: 60.4 ± 8.0	*T*1: 5.1 ± 4.1; *T*2: 3.5 ± 3.0; *C*: 4.6 ± 3.0 (yrs)	Fu Fang Nan Xing Zhi Tong patch versus placebo; Shang Shi Zhi Tong patch versus placebo for the treatment of 7 days	Pain, stiffness, and physical function; TCM syndrome	Fu Fang Nan Xing Zhi Tong patch leading to one withdrawal; 4 cases of rash, itching, slightly damaged skin, or erythema in two patches, respectively; no AEs were identified in placebo

Wang, 2006 [58]	42/40 (for all)	45~70 (for all)	1~18 (median = 7) (for all)	Xiao Tong patch versus diclofenac ointment for the treatment of 7 days	Global effectiveness rate	One case of mild local inflammation in treatment group (1/41; 2.44%); three cases of local allergic dermatitis found in control group (3/41; 7.32%)

Zhang, 2008 [20]	80 (for all)	Not reported	Not reported	Zhi Tong Tou Gu patch versus diclofenac ointment for the treatment of 28 days	Global effectiveness rate	Six cases exited the trial because of local allergy (not reported in which group)

Zheng, 2006 [23]	*T*: 12/24; *C*: 9/27	*T*: 51.06 ± 6.6; *C*: 52.78 ± 7.1	6 m~7 yrs (for all)	Qing Peng patch versus diclofenac ointment for the treatment of 21 days	Global effectiveness rate and pain	No AEs were identified

Values are the number (frequency or percentage). *T*: intervention group; *C*: control group.

**Table 2:** Characteristics of TCHPs for OA in included CCTs.

First author (year)	No. (M/F)	Age (yrs)	Disease duration	Eligibility criteria	Comparisons	AEs	Comparability at baseline
Liu, 2004 [42]	*T*: 40/50; *C*: 39/47	*T*: 68.5; *C*: 64.5	1 m~20 yrs (for all)	Unclear	Self-prescribed herbal patch versus sodium hyaluronate for the treatment of 5 weeks	No AEs were identified	Unclear

Cheng, 2009 [44]	238/122 (for all)	54.8 (for all)	2 m~20 yrs (for all)	The standard of TCM syndrome diagnostic and efficacy	San Huang patch versus Gu Tong patch for the treatment of 12 days	Unclear	Yes

Dong, 2007 [46]	*T*: 17/25; *C*: 14/22	*T*: 65.3; *C*: 68.2	*T*: 3.2; *C*: 3.4	ACR	Shu Jin patch versus Zhi Tong Xiao Yan patch for the treatment of 12 days	No AEs were identified	Yes

Feng*, 2006 [43]	*T*: 13/23; *C*: 15/18	Not reported	*T*: 50 ± 10; *C*: 50 ± 9	Hemigou	Gu Ci patch versus one control patch for the treatment of 9 days	No AEs were identified	Unclear

Kuang, 2010 [12]	*T*: 17/31; *C*: 18/28	*T*: 50.4 ± 8.53; *C*: 49.42 ± 9.47	*T*: 1.83 ± 0.35; *C*: 1.92 ± 0.47	ACR and clinical research guidelines of traditional Chinese patent drug	Zhong Tong Xiao Babu patch versus Zhong Tong Xiao patch for the treatment of 10 days	Unclear	Yes

Liu, 2008 [49]	122/238 (*T*: 260; *C*: 100; for all)	54.8 (for all)	2 m~50 yrs (for all)	ACR	Hei Hu patch versus Qian Shan Huo Xue patch for the treatment of 5 weeks	*T*: redness, oozing, purulent or itching, rash mentioned; *C*: fewer people of itching	Yes

Wang, 2005 [45]	*T*: 48; *C*: 16 (for all)	16~72 yrs (for all)	1~20 yrs (for all)	ACR	Zhen Tong Xiao Yan patch versus Fu Fang Nan Xing Zhi Tong patch for the treatment of 28 days	Unclear	Unclear

Wen, 2008 [34]	*T*: 13/39; *C*:14/40	*T*: 48~75 yrs; *C*: 47~72 yrs	*T*: 3 m~5 yrs; *C*: 47~74 yrs	ACR and clinical research guidelines of traditional Chinese patent drug	Xi Tong Kang patch versus Tong Luo Qu Tong pacth for the treatment of 28 days	Unclear	Yes

Xu, 2000 [48]	*T*: 31/65 (105); *C*: 20 (25)	*T*: 62.3; *C*: not reported	6 cases less than 1 year; 32 cases between 1 and 3 yrs; 28 cases more than 3 yrs	ACR	Fu Fang San sheng patch versus Zhuang Gu Guan Jie pill	2 cases showed local skin itching within 48 h after patching, which disappeared after a day by the discontinuation, but not affecting patching	Unclear

Zhang, 2010 [50]	*T*: 10/26; *C*: 13/23	*T*: 48.6; *C*: 51	*T*: 1 m~3 yrs; *C*: 1.5 m~3 yrs	ACR and clinical research guidelines of traditional Chinese patent drug	Gu Ci patch versus She Xiang Zhuang Gu patch for the treatment of 10 days	No AEs were identified	Unclear

Zhang, 2010 [33]	*T*: 18/36; *C*: 13/23	*T *: 45~85; *C *: 45~80	T: 3 m~5 yrs; C: 3 m~5 yrs	COA and the standard of TCM syndrome diagnostic and efficacy	Wen Tong patch versus Tong Luo Qu Tong patch for the treatment of 28 days	Unclear	Yes

Zhao, 2007 [47]	52/60 (for all)	10 cases (15 m~30 yrs); 20 cases (31~45 yrs); 35 cases (45 m~60 yrs); 47 cases more than 61 yrs	30 cases (6 m~3 yrs); 37 cases more than 10 yrs	The standard of TCM syndrome diagnostic and efficacy	Gu Bi Tong patch versus Fu Fang Nan Xing zhi Tong patch with 1-year follow-ups	Unclear	Unclear

*Three-arm study; Gu Ci patch versus control patch was selected.

**Table 3:** Effect estimates of TCHPs compared with diclofenac ointment or placebo for OA.

First author (year)	Effect estimate (95% CI)	Comparisons
Local pain relief		
Liu, 2004 [28]^#^	MD −1.14 (−3.56, 1.28)	Self-prescribed herbal patch versus diclofenac ointment
Wang, 2010 [21]	MD 0.00 (−1.09, 1.09)	Huo Xue Hua Yu patch versus diclofenac ointment
Wang, 2012 [4]*	MD −1.44 (−1.69, −1.19)	Fu Fang Nan Xing Zhi Tong patch versus placebo
Wang, 2012 [4]*	MD 1.08 (0.83, 1.33)	Shang Shi Zhi Tong patch versus placebo
Zheng, 2006 [23]	RR 0.50 (0.10, 2.56)	Qing Peng patch versus diclofenac ointment

Function of knee OA		
Liu, 2004 [28]^#^	MD −1.30 (−6.46, 3.86)	Self-prescribed herbal patch versus diclofenac ointment (function)
Wang, 2010 [21]	MD 0.06 (−0.29, 0.41)	Huo Xue Hua Yu patch versus diclofenac ointment (ROM)
Wang, 2010 [21]	MD 0.06 (−0.40, 0.52)	Huo Xue Hua Yu patch versus diclofenac ointment (flexion deformity)
Wang, 2012 [4]*	MD −0.42 (−0.47, −0.37)	Fu Fang Nan Xing Zhi Tong patch versus placebo (stiffness)
Wang, 2012 [4]*	MD −0.37 (−0.42, −0.32)	Shang Shi Zhi Tong patch versus placebo (stiffness)
Wang, 2012 [4]*	MD −2.61 (−3.01, −2.21)	Fu Fang Nan Xing Zhi Tong patch versus placebo (physical function)
Wang, 2012 [4]*	MD −2.97 (−3.38, −2.56)	Shang Shi Zhi Tong patch versus placebo (physical function)

Global effectiveness rate		
Lin, 2006 [25]^#^	RR 0.67 (0.13, 3.53)	Shang Ke Xiao Yan versus diclofenac
Long, 2006 [26]^#^	RR 1.87 (0.50, 7.01)	Shang Ke Hei Yao patch versus diclofenac ointment
Guan, 2010 [22]	RR 0.43 (0.12, 1.51)	Zhuang Gu Tong Bi patch versus diclofenac ointment
Zheng, 2006 [23]	RR 0.25 (0.03, 2.13)	Qing Peng patch versus diclofenac ointment
Zhang, 2008 [20]	RR 0.43 (0.14, 1.26)	Zhi Tong Tou Gu patch versus diclofenac ointment
Wang, 2006 [58]	RR 0.22 (0.05, 0.97)	Xiao Tong patch versus diclofenac ointment

Data was synthesized using MD with 95% CI for continuous outcomes or RR with 95% CI for binary outcomes; *there was no statistically significant difference between the intervention and control group in score reduction or global effectiveness rate (*P* > 0.05); ^#^noninferiority results.

**Table 4:** Effect estimates of TCHPs for OA about CCTs.

First author (year)	Treatment group (*n*/*N*)	Control group (*n*/*N*)	Effect estimate (95% CI)	Comparisons
Global effectiveness rate				
Liu, 2004 [42]	12/90	24/86	RR 0.48 (0.26, 0.89)	Self-prescribed herbal patch versus sodium hyaluronate
Cheng, 2009 [44]	3/42	3/21	RR 0.50 (0.11, 2.27)	San Huang patch versus Gu Tong patch
Dong, 2007 [46]	3/42	5/36	RR 0.51 (0.13, 2.00)	Shu Jin patch versus Zhi Tong Xiao Yan patch
Feng, 2006 [43]*	5/36 6/36	8/33 12/33	RR 0.48 (0.26, 0.89)^#^ RR 0.46 (0.19, 1.08)^§^	Gu Ci patch versus one control patch
Kuang, 2010 [12]	4/48	9/46	RR 0.43 (0.14, 1.29)	Zhong Tong Xiao Babu patch versus Zhong Tong Xiao patch
Liu, 2008 [49]	4/260	4/100	RR 0.38 (0.10, 1.51)	Hei Hu patch versus Qian Shan Huo Xue patch
Wang, 2005 [45]	1/48	4/18	RR 0.09 (0.01, 0.78)	Zhen Tong Xiao Yan patch versus Fu Fang Nan Xing Zhi Tong patch
Wen, 2008 [34]	5/52	11/54	RR 0.47 (0.18, 1.27)	Xi Tong Kang patch versus Tong Luo Qu Tong patch
Xu, 2000 [48]	7/105	5/20	RR 0.27 (0.09, 0.76)	Fu Fang San sheng patch versus Zhuang Gu Guan Jie pill
Zhang, 2010 [50]	6/36 (5d) 4/36 (10d)	9/36 (5d) 7/36 (10d)	RR 0.67 (0.26, 1.68) RR 0.57 (0.18, 1.78)	Gu Ci pacth versus She Xiang Zhuang Gu patch
Zhang, 2010 [33]	6/54	12/54	RR 0.50 (0.20, 1.24)	Wen Tong patch versus Tong Luo Qu Tong patch
Zhao, 2007 [47]	7/62	15/50	RR 0.38 (0.17, 0.85)	Gu Bi Tong patch versus Fu Fang Nan Xing Zhi Tong patch

Data was synthesized using RR with 95% CI; *three-arm study, knee pain, and ROM about “Gu Ci” patch versus control patch were obtained; ^#^for knee pain; ^§^for ROM.

**Table 5:** Top 20 most frequently used herbs from 86 kinds of TCHPs.

Herbs	Freq. (*n*)	Percentage (%)
Chuan Wu (*Radix Aconiti*)	37	43.02
Cao Wu (*Radix Aconiti Kusnezoffii*)	34	39.53
Mo Yao (*Myrrha*)	33	38.37
Ru Xiang (*Olibanum*)	32	37.21
Dang Gui (*Radix Angelicae*)	29	33.72
Bing Pian (*Borneolum Syntheticum*)	29	33.72
Chuan Xiong (*Rhizoma Ligusticum chuanxiong*)	28	33.56
Bai Zhi (*Dahuricae*)	25	29.07
Wei Ling Xian (*Radix Clematisdis*)	24	27.91
Tian Nan Xing (*Rhizoma Arisaematis*)	22	25.58
Xi Xin (*Herba Asari*)	21	24.42
Ma Qian Zi (*Semen Strychni*)	21	24.42
Hong Hua (*Flos Carthami*)	20	23.25
Niu Xi (*Radix Achyranthis Bidentatae*)	19	22.09
She Xiang (*Moschus*)	18	20.93
Zhang Nao (*Camphora*)	18	20.93
Du Huo (*Angelicae Pubescentis*)	17	19.77
Da Huang (*Radix et Rhizoma Rhei*)	17	19.77
Rou Gui (*Cortex Cinnamomi*, 15)	15	17.44
Xu Duan (*Radix Dipsaci*)	15	17.44

Values are the number (frequency or percentage).

**Table 6:** Herbs and actions of TCHPs.

Categories of effectiveness	
Expelling wind-cold and eliminating dampness medicinal (accumulated no.: 37; accumulated freq.: 286)	

Chuan Wu (*Radix Aconiti*, 37), Cao Wu (*Radix Aconiti Kusnezoffii*, 34), Bai Zhi (*Radix Angelicae Dahuricae*, 25), Wei Ling Xian (*Radix Clematidis*, 24), Xi Xin (*Herba Asari*, 21), Du Huo (*Angelicae Pubescentis*, 17), Ma Huang (*Herba Ephedra*, 15), Mu Gua (*Fructus Chaenomelis*, 14), Tou Gu Cao (*Herba Speranskiae Tuberculatae*, 14), Gui Zhi (*Ramulus Cinnamomie, Cassia Twig*, 12), Qiang Huo (*Rhizoma seu Radix Notopterygii*, 12), Fang Feng (*Radix Saposhnikoviae*, 9), Shen Jin Cao (*Herba Lycopodir*, 5), Qing Feng Teng (*Caulis Sinomenii seu Diploclisiae*, 4), Sang Ji Sheng (*Loranthi*, 3), Song Jie (*Lignum Pini Nodi*, 3), Hai Feng Teng (*Caulis Piperis Futokadsuae*, 3), Ge Gen (*Radix Puerariae*, 3), Wu Shao She (*Zaocys dhumnades*, 3), Xue Shang Yi Zhi Hao (*Radix Aconiti brachypodi*, 3), Xu Chang Qing (*Radix Cynaachi Paniculati*, 3), Du Yi Wei (*Radix Lamiophlomidis rotatae*, 3), Man Jing Zi (*Fructus viticis*, 2), Mu Bie Zi (*Semen Momordicae*, 2), Hai Tong Pi (*Cortex Erythrinae*, 2), Xi Qian Cao (*Herba Siegesbeckiae*, 2), others (11).	

Blood-activating and stasis-resolving medicinal (accumulated no.: 27; accumulated freq.: 268)	

Mo Yao (*Myrrha*, 33), Ru Xiang (*Olibanum*, 32), Chuan Xiong (*Rhizoma Ligusticum chuan xiong*, 28), Ma Qian Zi (*Semen Strychni*, 21), Hong Hua (*Flos Carthami*, 20), Niu Xi (*Radix Achyranthis Bidentatae*, 19), She Xiang (*Moschus*, 18), Xue Jie (*Sanguis Draconis*, 14), Tu Bie Chong (*Eupolyphaga seu Steleophaga*, 11), San Qi (*Radix Notoginseng*, 11), Chuan Shan Jia (*Squama Manitis*, 11), Jiang Huang (*Rhizoma Curcumame Longae*, 10), Tao Ren (*Semen Persicae*, 6), E. Zhu (*Rhizoma Curcumae*, 5), San Ling (*Rhizoma Sparaganii*, 5), Yan Hu Suo (*Rhizoma Corydalis*, 5), Pu Huang (*Pollen Typhae*, 4), Ji Xue Teng (*Caulis Spatholobi*, 3), Wu Ling Zhi (*Faeces Trogopterori*, 2), Su Mu (*Lignum Sappan*, 2), Ban Mao (*Radix Saccharri arundinacei*, 2), and others (6).	

Tonic medicinal (accumulated no.: 24; accumulated freq.: 107)	

Dang Gui (*Radix Angelicae*, 29), Xu Duan (*Radix Dipsaci*, 15), Gu Sui Bu (*Rhizoma Drynariae*, 11), Wu Jia Pi (*Cortex Acanthopanacis*, 10), Du Zhong (*Cortex Eucommiae*, 7), Bu Gu Zhi (*Fructus Psoraleae*, 5), Huang Qi (*Radix Astragali*, 4), Gou Ji (*Rhizoma Cibotii*, 4), Shu Di Huang (*Radix Rehmanniae Preparata*, 3), Bai Shao (*Radix Paeoniae*, 3), Yin Yang Huo (*Herba Epimedii*, 3), and others (13).	

Heat-clearing medicinal (accumulated no.: 22; accumulated freq.: 92)	

Da Huang (*Radix et Rhizoma Rhei*, 17), Chi Shao (*Radix Paeoniae Rubra*, 10), Zhi Zi (*Fructus Gardeniae*, 7), Dan Shen (*Radix Salviae Miltiorrhizae*, 7), Qin Jiao (*Radix Gentianae Macrophyllae*, 7), Huang Bo (*Cortex Phellodendri*, 6), Huang Qin (*Radix Scutellariae*, 5), Fang Ji (*Radix Stephaniae Tetrandrae*, 5), Hua Shi (*Talcum*, 4), Ling Qiao (*Fructus Forsythiae*, 4), Di Long (*Pheretima*, 3), Mang Xiao (*Natrii Sulfas*, 3), Shi Gao (*Gypsum Fibrosum*, 2), Tian Hua Feng (*Radix Trichosanthis*, 2), Pu Gong Ying (*Herba Taraxaci*, 2), Chuan Shan Long (*Dioscorea nipponica Makino*, 2), and others (6).	

Phlegm-eliminating and damp-draining medicinal (accumulated no.: 19; accumulated freq.: 63)	

Tian Nan Xing (*Rhizoma Arisaematis*, 22), Bai Jie Zi (*Semen Sinapis*, 13), Zao Jiao (*Fructus Gleditsiae*, 4), Cang Zhu (*Rhizoma Atractylodis*, 3), Yi Yi Ren (*Semen Coicis*, 3), Jiang Can (*Bombyx Batryticatus*, 3), Huo Xiang (H*erba Pogostemonis*, 2), Hai Zao (*Sargassum, Seaweed*, 2), and others (11).	

Interior-waring medicinal (accumulated no.: 7; accumulated freq.: 40)	

Rou Gui (*Cortex Cinnamomi*, 15), Ding Xiang (*Flos Caryophylli*, 8), Gan Jiang (*Rhizoma Zingiberis*, 6), Xiao Hui Xiang (*Fructus Foeniculi*, 4), Gao Liang Jiang (*Rhizoma Alpiniae Officinarum*, 3), Hua Jiao (*Pericarpium*, 3), and others (1).	

Qi-regulating medicinal (accumulated no.: 5; accumulated freq.: 8)	

Xiang Fu (*Rhizoma Cyperi*, 3), Mu Xiang (*Radix Aucklandiae*, 2), and others (3).	

Others (accumulated no.: 38; accumulated freq.: 117)	

Bing Pian (*Borneolum Syntheticum*, 29), Zhang Nao (*Camphora*, 18), Qian Dan (*Componere Hydrargyrum*, 9), Quan Xie (*Scorpio*, 8), Xiong Huang (*Realgar*, 6), Bo He (*Herba Menthae*, 4), Zi Jin Pi (*Cortex Cercis Chinensis*, 3), Teng Huang (*Garcinia morella Desv*, 3), A. Wei (*Resina Ferulae*, 3), Ji Dou (*Herba Oxytropis chiliophyllae*, 2), Chan Su (*Venenum Bufonis*, 2), Yang Jin Hua (*Flos Daturae*, 2), Ma Ren (*Semen Cannabis*, 2), Sha Jiang (*Rhizoma Kaempferiae*, 2), and others (24).	

Values are the number (frequency). Herbal name presented only when the value is above 1.

**Table 7:** Detailed AEs of TCHPs for OA after 3-4-week follow-ups.

First author (year)	Intervention group			Control group		
	Patches	Incidence	AEs	Patches	Incidence	AEs
Cao*, 2002 [84]	Qu Tong	11.49% (10/87)	Skin allergy	Gu Tong	Not stated	Not stated

Du, 1997 [53]	Ji Li Huo Xue	10.00% (6/60)	Erythema after 4-5 days; itching in the location of patch	Dong Fang Huo Xue	Not stated	Not stated

Guo, 2008 [36]	Xiong Zhi Tong Xiao	0.66% (1/152)	Itching	Tong Luo Qu Yu	2% (1/50)	Itching

Hao, 1999 [70]	Feng Shi Shang Tong	6.67% (4/60)	Contact dermatitis and exit, itchy skin	NA	NA	NA

Hao, 1999 [64]	Fu Fang Ling Zhi	6.67% (4/60)	Contact dermatitis and exit, itching after 48 hours	NA	NA	NA

Li, 2009 [66]	Yao Tong Ning	2.08% (1/48)	Terminated with locally severe blister	NA	NA	NA

Li, 2009^§^ [71]	Ba Wei	No data	Local discomfort, burning sensation, itching, or rash	Shang Shi Zhi Tong	No data	Local discomfort, burning sensation, itching, or rash

Li, 2005 [67]	Mei Pu Zheng Gu	No data	Rash, blister, and itching	NA	NA	NA

Lin, 2006 [25]	Shang Ke Xiao Yan	No data	No significant allergic reaction	Diclofenac sodium tablets	No data	Not stated

Liu, 2011 [72]	Xiao Tong	8.89% (4/45)	Rash, burning sensation, and itching	Gu Tong	6.67% (3/45)	Rash

Liu, 2008 [49]	Hei Hu	No data	Redness, oozing, purulent or itching, and rash	Qian Shan Huo Xue	Few people	Local itching

Ren, 1998 [68]	Gu Ci Ting	No data	Skin redness and blister	NA	NA	NA

Su, 2010 [73]	Jie Gu	6.41% (5/78)	Gastrointestinal discomfort, unsatisfied	Glucosamine sulfate	5.71% (4/70)	Gastrointestinal discomfort, unsatisfied

Tao, 2005 [69]	Xiao Zhong Zhi Tong	No data	Skin redness, itching, and blister	NA	NA	NA

Wang, 2002 [85]	Fu Fang Yan Tong Ning	1.00% (2/200)	Rash and itching	Gou Pi	4.00% (4/100)	Rash and itching

Wang, 2012 [4]	Fu Fang Nan Xing and Shang Shi Jie Tong	7.50% (9/120)	Rash, itching, and erythema; contact dermatitis	Placebo	0.00% (0/30)	None

Wang, 2008 [57]	Feng Shi Gu Tong	2.00% (2/100)	Rash	Gou Pi	4.44% (4/90)	Allergic reactions

Xu, 2000 [48]	Fu Fang San Sheng	2.64% (2/76)	Itchy skin after 48 hours	Zhuang Gu Guan Jie pill	0.00% (0/20)	None

Yang, 1999 [30]	Gu Zheng Sheng Zheng	4.00% (2/50)	Rash	Gu Yong Ling liniment	8.00% (2/25)	Mild rash, flushing

Wu, 2005 [41]	Blistering therapy	50.00% (15/30)	Pain and itching rash	He Luo Zhi Tong	6.67% (2/30)	Allergic reactions

Zeng, 2010 [74]	Tong Yu	8.00% (2/25)	Mild stomach discomfort and mild nausea in the beginning	Xiao Tong	Not stated	Not stated

Zhang, 2008 [20]	Zhi Tong Tou Gu	12.24% (6/49)	Skin rash, erythema, and so forth.	Diclofenac ointment	24.00% (6/25)	Skin rash, erythema, and so forth.

Zhang, 2005 [35]	She Xiang Tong Bi Ba Bu	5.22% (6/115)	Rash and itching	Tong Luo Qu Gu	7.96% (9/113)	Itching, flushing, swelling, and so forth.

Pan, 2008 [32]	Gu Tong Ning	5.65% (19/336)	Redness and itching	Gu Tong	8.93% (10/112)	Redness and itching

Zhang, 2011 [86]	Qu Yu Zhi Tong	No data	Few AEs	Sodium hyaluronate	Not stated	Not stated

Zhou, 2003 [87]	Wei Ling Xian	Few patients	Blistering	NA	NA	NA

Values are based on identified data. *No specific data reported for each AEs. ^§^Lower AEs in intervention group. ^#^A special therapy mainly for blistering. NA: not applicable.

## References

[1] Felson DT (2006). Osteoarthritis of the knee. *The New England Journal of Medicine*.

[2] Debbi EM, Agar G, Fichman G (2011). Efficacy of methylsulfonylmethane supplementation on osteoarthritis of the knee: a randomized controlled study. *BMC Complementary and Alternative Medicine*.

[3] Chinese Medical Association of Orthopaedics (2007). Osteoarthritis guidelines of diagnosis and treatment. *Chinese Journal of Joint Surgery*.

[4] Wang X, Cao Y, Pang J (2012). Traditional Chinese herbal patch for short-term management of knee osteoarthritis: a randomized, double-blind, placebo-controlled trial. *Evidence-Based Complementary and Alternative Medicine*.

[5] Long L, Soeken K, Ernst E (2001). Herbal medicines for the treatment of osteoarthritis: a systematic review. *Rheumatology*.

[6] Ernst E (2011). Herbal medicine in the treatment of rheumatic diseases. *Rheumatic Disease Clinics of North America*.

[7] Ernst E, Posadzki P (2011). Complementary and alternative medicine for rheumatoid arthritis and osteoarthritis: an overview of systematic reviews. *Current Pain and Headache Reports*.

[8] Xu YP, Xie LM, Wang WY (2012). Meta-analysis on external use of tradition Chinese medicine (TCM) in treating knee osteoarthritis. *China Journal of Chinese Materia Medica*.

[9] Hochberg MC, Altman RD, April K (2012). American college of rheumatology 2012 recommendations for the use of nonpharmacologic and pharmacologic therapies in osteoarthritis of the hand, hip and knee. *Arthritis Care and Research*.

[10] Cao Y, Zhan H, Pang J (2011). Individually integrated traditional Chinese medicine approach in the management of knee osteoarthritis: study protocol for a randomized controlled trial. *Trials*.

[11] Hsu WH, Ho TJ, Huang CY (2010). Chinese medicine acupoint herbal patching for allergic rhinitis: a randomized controlled clinical trial. *American Journal of Chinese Medicine*.

[12] Kuang JJ (2010). Zhong Tong Xiao Papua patch for the treatment of osteoarthritis of the knee 48 cases. *Hunan Journal of Chinese Medicine*.

[13] Du XH (2010). Clinical observation of Gu Bi Xi Tong Papua patch for the topical treatment of knee osteoarthritis. *Journal of New Chinese Medicine*.

[14] Hu C, Chen RM, Yin SM (2009). Observation on analgesic effect and mechanism of Fufang Nanxing Zhitong Gao. *Journal of Nanjing Niversity of Traditional Chinese Medicine*.

[15] Bian HM, Yu JH, Jiang M, Sun L (2007). Anti-inflammatory effect of Fufang Nanxing Zhitong Gao. *Pharmacology and Clinics of Chinese Materia Medica*.

[16] Hinman RS, Crossley KM, McConnell J, Bennell KL (2003). Efficacy of knee tape in the management of osteoarthritis of the knee: blinded randomised controlled trial. *British Medical Journal*.

[17] China State Administration of Traditional Chinese Medicine (2011). *Clinical Path of Traditional Chinese Medicine 22 Professional 95 Diseases*.

[18] Gao XM (2002). *Traditional Chinese Medicine*.

[19] Xutian S, Cao D, Wozniak J, Junion J, Boisvert J (2012). Comprehension of the unique characteristics of traditional Chinese medicine. *American Journal of Chinese Medicine*.

[20] Zhang JG, Fu DM, Yang CL (2008). The clinical research about the therapy of bone arthritis of knee joint by Zhitong Tougu Ointment. *China Medical Herald*.

[21] Wang P, Gu EP, Cao HY (2010). Clinical observation of Huo Xue Hua Yu patch for the treatment of early and mid-osteoarthritis of the knee. *Jilin Traditional Chinese Medicine*.

[22] Guan ZY (2010). Zhuanggu Tongbi cream in treatment of knee osteoarthritis clinical research. *Clinical Journal of Chinese Medicine*.

[23] Zheng YX, Zhan HS, Zhang H, Niu SG, Zhuang ZJ (2006). Qi-zheng Qing-peng slurry for treatment of the knee osteoarthritis: a randomized, controlled clinical research. *China Journal of Orthopaedics and Traumatology*.

[24] Sun XL (2009). Huangbo Wuwei patch for the treatment of knee osteoarthritis 90 cases. *Modern Traditional Chinese Medicine*.

[25] Lin L (2006). Clinical observation of Shang Ke Xiao Yan patch for the topical treatment of the cold-damp stasis of knee osteoarthritis. *Morden Medicine Drug and Health*.

[26] Long CQ (2006). Clinical observation of Guan Jie Yan patch for the topical treatment of knee osteoarthritis. *Modern Journal of Integrated Traditional Chinese and Western Medicine*.

[27] Liao J, Lv FM, Meng QC (2010). Clinical efficacy of Re Yun I recipe to treat type of Yang Xu Han Nin in knee osteoarthritis. *Xinjiang Medical University*.

[28] Liu JP, Yang MH, Qiu Y, Huang TJ (2004). Observed clinical efficacy of traditional Chinese medicine for the topical treatment of osteoarthritis of the knee. *Xinjiang Traditional Chinese Medicine*.

[29] Shen J, Chen JY, Yun XL, Zhu QQ, Li YX (2011). Clinical comparative study on self-made Tongluo Qing Bi patch for the treatment of knee osteoarthritis. *Journal of Guiyang College of Traditional Chinese Medicine*.

[30] Yang YQ (1999). Clinical study of Gu Zeng Sheng Zheng Tong for the treatment of osteoarthritis. *Traditional Chinese Drug Research & Clinical Pharmacology*.

[31] Long ZQ (2006). Clinical observation of Guan Jie Yan patch for the topical treatment of knee osteoarthritis. *Modern Journal of Integrated Traditional Chinese and Western Medicine*.

[32] Pan MC (2008). Gu Tong Ning patch for the treatment of knee osteoarthritis 216 cases. *China Foreign Medical Ttreatment*.

[33] Zhang Y, Xin YM (2010). Clinical observation of homemade Wentong patch for the topical treatment of osteoarthritis of the knee. *Chinese Manipulation & Rehabilitation Medicine*.

[34] Wen H, Zhao WH, Li XC (2008). Clinical observation of Xi Tong Gao for the treatment of osteoarthritis of the knee. *Jilin Journal of Traditional Chinese Medicine*.

[35] Zhang Q, Xiao J, Dun L (2005). Clinical study of Shexiang Tongbi Pupua patch for the treatment of the symdrone of liver and kedney defficious and stens-muslcuar stasis of oateoarthritis. *Chinese Journal of Clinical Pharmacology and Therapeutics*.

[36] Guo JF, Du ZQ, Bao TZ (2008). Clinical study of Xiong Zhi Tong Yu Xiao San Tie on knee pain (blood stasis syndrome) induced by osteoarthritis. *World Journal of Integrated Traditional and Western Medicine*.

[37] Ding GS, Shen QR, Xie HY (2008). Preparation and clinical observation of Kang Wei Tong Bi patch. *Chinese Anchive of Traditional Chinese Medicine*.

[38] Tan ZA, Li JW (2010). Clinical study of Jinghuang for knee plaster on external application for knee osteoarthritis. *Journal of Traditional Chinese Medicine University of Hunan*.

[39] Xu EP, Li GL, Yang QH, Jang SQ (2002). Gu Bi patch for the treatment of knee osteoarthritis 78 cases. *New Traditional Chinese Medicine*.

[40] Bai SC, Wang ZG, Zhang FJ, Li H, Zeng LH (2004). Clinical research of Gu Tong patch in curing proliferative arthritis of knee joint. *Chinese Journal of Traditional Medicine Traumatology & Orthopedics*.

[41] Wu JX, Huang CX, Lin JY, Tang ZL (2005). Clinical observation of Xi Tong Ning patch for the topical treatment of knee osteoarthritis. *China Journal of Orthopaedics and Traumatology*.

[42] Liu JP, Yang MH, Qiu Y, Huang TJ (2004). Observed clinical efficacy of traditional Chinese medicine for the topical treatment of osteoarthritis of the knee. *Xinjiang Traditional Chinese Medicine*.

[43] Feng KH (2006). Clinical efficacy judgment of Gu Ci patch for the treatment of osteoarthritis of the knee. *Health Vocational Education*.

[44] Cheng YF, Gu SF, Liu M (2009). San Huang patch for the topical treatment of knee osteoarthritis 63 cases. *Shenzhen Journal of Integrated Traditional and Western Medicine*.

[45] Wang JM, Wu LW (2005). Zheng Tong Xiao Yan patch for the treatment of osteoarthritis 66 cases. *Heilongjiang Journal of Traditional Chinese Medicine*.

[46] Dong Z (2007). Clinical research of Shujin patch for the treatment of osteoarthritis of the knee. *Journal of Fujian College of Traditional Chinese Medicine*.

[47] Zhao AL (2007). Report of 112 cases about Gu Bi Tong patch for the topical treatment of bone and joint disease. *Zhejiang University of Traditional Chinese Medicine*.

[48] B, Xu, Lu JF (2000). Clinical research of Fu Fang San Sheng patch for the treatment of knee ostoarthritis. *Clinical Medicine*.

[49] Liu SG, Jiang YG (2008). Hei Hu patch for the treatment of knee osteoarthritis 260 cases. *Shanxi Journal of Traditional Chinese Medicine*.

[50] Zhang L, Dong L, Zhao DZ, Zhang DJ (2010). Observation on treating osteoarthritis pain with Gu Ci Xiao cataplasm. *Chinese Journal of the Practical Chinese With Modern Medicine*.

[51] Yan JL, Cheng FR, Wang XJ, Tang JY, Wu BG, Ling YX (2000). Clinical observation of Le E acupoint patch for the treatment of knee osteoarthritis 50 cases. *Chinese Journal of Traditional Medical Science and Technology*.

[52] Zhou XH, Lin D, Meng WC, Li F, Lin HS (2000). Clinical study of Gu Ci Ning patch for the treatment of deenerative joint disease 100 cases. *Guangzhou University of Traditional Chinese Medicine*.

[53] Du XL, Zhang H (1997). Clinical research of Ji Li Huo Xue patch for the treatment of knee osteoarthritis. *Journal of Shandong University of Traditional Chinese Medicine*.

[54] Shi B, Li G (2006). Clinical study on Fu Fang Huo Xue patch for the topical treatment of osteoarthritis of the knee (0–II period). *Journal of External Therapy of Traditional Chinese Medicine*.

[55] Yin H, Ma Y, Wang JW (2011). Patching therapy for the treatment of knee osteoarthritis 60 cases. *Traditional Chinese Medicine*.

[56] Chen YQ, Wu JH, Yao HM, Lv JY, Sun YL, Yin SM (2010). Clinical study on compound nanxing pain paste in treating 249 cases of osteoarthritis of cold dampness and blood stasis. *Shanghai Journal of Traditional Chinese Medicine*.

[57] Wang YG, Wei DY, Wang FC, Liang XL (2008). Feng Shi Gu Tong patch for the treatment of osteoarthritis. *China's Naturopathy*.

[58] Wang YY, Liu HJ (2006). Clinical observation of Xiao Tong patch for knee osteoarthritis 41 cases. *Chongqing Medicine*.

[59] Shao SJ, Ji LX, Liu N, Liu SM, Tu Q, Shi XM, Dai XM, Wang J (2009). Syndrome differentiation of limbs and meridians. *Internal Medicine of Traditional Chinese Medicine*.

[60] Wang HQ, Wang YY, Li WX, Zou YC, Liu B (2012). Clinical efficacy of compound Nanxing Zhitong Ointment on agonizing-arthragia type knee osteoarthritis. *Journal of New Chinese Medicine*.

[61] Wu JX, Huang CX, Lin JY, Tang ZL (2005). Clinical observation of Xi Tong Ning patch for the topical treatment of knee osteoarthritis. *China Journal of Orthopaedics and Traumatology*.

[62] Zhou XS, Yi DB, Zhu JY, Zhou K (2006). Preparation and clinical appliance of Wei Ling Xian patch. *Chinese Journal of Hospital Pharmacy*.

[63] Wang HJ, Fan XZ (1998). Clinical observation Gu Zhi Zhi Tong patch for the treatment of bi-arthralgia of cervical spondylos. *Practical Traditional Chinese Medicine*.

[64] Hao FT, Jiang ZG, Tian CY (1999). Effeciacy observation of Fufang Lingzhi patch for the treatment of osteoarthritis of the knee. *Research of Traditional Chinese Medicine*.

[65] Yin YL, Wang XQ, Li QH (1999). Ru Gui patch for the topical treatment of knee osteoarthritis 100 cases. *Journal of Henan College of Traditional Chinese Medicine*.

[66] Li HM, Liu FL, Guo SQ (2009). Yao Tong Ning patch for the topical treatment of osteoarthritis of the knee 48 cases. *Journal of External Therapy of Traditional Chinese Medicine*.

[67] Li MP, Han QB, Wang QQ, Zhang YF, Ma QS (2005). Homemade Mei Po Zheng Gu patch for the treatment of bone hyperplasia 132 cases. *Clinical Medicine of Traditional Chinese Medicine*.

[68] Ren XP (1998). Gu Ci Ting patch for the treatment of bone hyperplasia in 528 patients. *Shanxi Journal of Traditional Chinese Medicine*.

[69] Tao HJ (2005). Young's Xiao Zhong Zhi Tong patch for the treatment of bone hyperplasia of lumbar spine 200 cases. *Jangsu Journal of Traditional Chinese Medicine*.

[70] Hao FT, Hao QL, Jiang ZG, Xiu GW, Yan CS, Yan T (1999). Effeciacy observation of Feng Shi Shang Tong patch for the treatment of osteoarthritis of the knee. *Journal of External Therapy of Traditional Chinese Medicine*.

[71] Li M, Xiu PF, Zhu CG (2009). Ba Wei patch for the treatment of knee osteoarthritis 80 cases. *Shanxi Journal of Traditional Chinese Medicine*.

[72] Liu G (2011). Efficacy observation of Qizheng Xiaotong patch for the treatment of osteoarthritis of the knee. *Contemporary Medicine*.

[73] Su LC (2010). Clinical observation of Jiegu patch for the treatment of osteoarthritis of the knee effusion. *Chinese Medicine Morden Distance Education of China*.

[74] Zeng CH, Su XH, Zhao JL (2010). Tong Yu patch for the treatment of osteoarthritis of the knee 25 cases. *Hunan Journal of Traditional Chinese Medicine*.

[75] Liu GD (2010). *Systematic review of oral tradition Chinese medicine (TCM) in treating knee osteoarthritis [M.S. thesis]*.

[76] Moher D, Hopewell S, Schulz KF (2010). CONSORT 2010 explanation and elaboration: updated guidelines for reporting parallel group randomised trials. *Journal of Clinical Epidemiology*.

[77] Deng ZJ (2003). Damp-draining formulae. *Traditional Chinese Medical Formulae*.

[78] Pan JQ, Xiao LY, Zhang D (2003). Immunosuppressive, antioxidant, anti-inflammatory and analgesic effect of Xiao Huo Luo Dan(pill). *Guangdong Pharmaceutical*.

[79] Wei D (2010). Xiao Huo Luo Dan(pill) for the treatment of early and mid-knee osteoarthritis 120 cases. *The Journal of Traditional Chinese Orthopaedics and Traumatology*.

[80] Deng ZJ (2003). Wind-expelling formulae. *Traditional Chinese Medical Formulae*.

[81] Teekachunhatean S, Kunanusorn P, Rojanasthien N (2004). Chinese herbal recipe versus diclofenac in symptomatic treatment of osteoarthritis of the knee: a randomized controlled trial [ISRCTN70292892]. *BMC Complementary and Alternative Medicine*.

[82] Li ZR, Lv CQ (2011). Du Huo Ji Sheng Tang (decoction) for the treatment of knee osteoarthritis 36 cases by oral administration and external washing. *Shanxi Journal of Traditional Chinese Medicine*.

[83] Wang WL, Ye JX, Liu XX, Shen FE, Li P, Zhang YY (2011). Clinical observation of Du Huo Ji Sheng Tang(decoction) for the treatment of knee osteoarthritis 66 cases by oral administration and external washing. *Journal of Fujian University of Traditional Chinese Medicine*.

[84] Cao Y, Yao JP, Dong JN, Lin YH, Gao JZ (2002). Clinical observation of Qu Tong patch for the treatment of degenerative knee joint disease. *Liaoning Journal of Traditional Chinese Medicine*.

[85] Wang C (2002). Clinical research of Fu Fang Yan Ning patch for the treatment of bone hyperplasia. *Chinese Journal Traditional Medicine Traumatology & Orthopedics*.

[86] Zhang Y, Xie YL, Zhang QT, Ma T, Li LJ (2011). Clinical observation of Qu Yu Zhi Tong patch for the treatment of osteoarthritis of the knee. *Inner Mongolia in Traditional Chinese Medicine*.

[87] Zhou XS, Yi DB (2003). Pharmacodynamical research on anti-inflammatory of Wei Ling Xian. *Chinese Journal of Clinical Medicine and Pharmacy*.

