# Supplementary material for: Effectiveness, Medication Patterns, and Adverse Events of Traditional Chinese Herbal Patches for Osteoarthritis: A Systematic Review
Source: Evid Based Complement Alternat Med. 2014 Jan 14;2014:343176. doi: 10.1155/2014/343176 (PMC3914464; doi:10.1155/2014/343176)
Supplement: Supplementary file 1 — Search process and risk of bias assessment of TCHPs. [file 343176.f1.docx]

**Appendix I** **Electronic database search.**

**PubMed**

1. exp "Clinical Trial [Publication Type]"/

2. exp "Randomized Controlled trials [Publication Type]"/

3. exp "Case Reports [Publication Type]"/

4. exp " placebo "/

5. exp "Control Groups "/

6. trial.ab,ti.

7. randomly.ab,ti.

8. or/#1-#7

9. Animals/

10. Humans/

11. #9 not (#9 and #10)

12. #8 not #11

13. exp Osteoarthritis/

14. exp Arthralgia/

15. exp Chronic Pain/

16. exp Musculoskeletal Pain/

17. exp Rheumatic Diseases/

18. or/#13-#17

19. exp Medicine, Chinese Traditional/

20. exp Medicine, Oriental Traditional/

21. exp Medicine, Traditional/

22. exp Drugs, Chinese Herbal/

23. exp Drugs, Nonprescription/

24. exp Complementary Therapies/

25. complementary medicine.tw.

26. alternative medicine.tw.

27. Chinese medicine.tw.

28. traditional Chinese medicine.tw.

29. Chinese herbal patch.tw.

30. Herbal patch.tw.

31. Chinese patch.tw.

32. Plaster.tw.

33. or/#19-#32

34. #12 and #18 and #33

**Ovid**

1.randomized controlled trial.pt.

2.controlled clinical trial.pt.

3.randomized.ab.

4.placebo.ab.

5.randomly.ab.

6.trial.ab.

7.groups.ab.

8. case reports.pt.

9. exp observational studies/

10. exp clinical studies/

11. OR/1-10

12. animals.ab.

13. humans.ab.

14.12 NOT 13

15. 11 NOT14

16. exp Knee/

17. knee joint.ab.

18.patellofemoral.ab.

19.tibiofemoral.ab.

20.OR/16–19

21. exp osteoarthritis/

22.degenerative disease.tw.

23.osteoarthr$.tw.

24.(degenerative adj2 arthritis).tw

25.OR/21–24

26. expTraditional Chinese Medicine/

27. complementary medicine.ab.

28. alternative medicine.ab.

29. herbal patch$.af.

30. plaster.tw.

31. OR/26–30

32. AND/11, 15,20,25,31

33. Remove duplicates

**CENTRAL**

1. MeSH descriptor Osteoarthritis, this term only

2. MeSH descriptor Arthralgia, explode all trees

3. MeSH descriptor Chronic Pain, explode all trees

4. MeSH descriptor Musculoskeletal Pain, explode all trees

5. (#1 or #2 or #3 or #4)

6. Chinese medicine in All Fields in all Cochrane products

7. alternative medicine in All Fields in all Cochrane products

8. complementary medicine in All Fields in all Cochrane products

9. nonprescription drugs in All Fields in all Cochrane products

10. traditional Chinese medicine in All Fields in all Cochrane products

11. Chinese herbal patch* in All Fields in all Cochrane products

12. Herbal patch* in All Fields in all Cochrane products

13. Chinese patch* in All Fields in all Cochrane products

14. Plaster in All Fields in all Cochrane products

15. (#6 or # 7 or #8 or #9 or #10 or #11 or #12 or #13 or #14)

16. (#5 and #15)

17."accession number" near pubmed

18. (#16 not #17)

**Sinomed** (formerly Chinese Biomedical Literature; CBM) (These search queries were adapted for **CNKI**)

1. exp Clinical Trial / all subheadings

2. exp Randomized Controlled trials / all subheadings

3. exp Random Allocation/ all subheadings

4. exp Multicenter Study / all subheadings

5. Random*

6. Control*

7. exp Case Reports / all subheadings

8. or/ #1-#7

9. exp Osteoarthritis / all trees and all subheadings

10. exp Osteoarthropathy / all trees and all subheadings

11. exp Arthralgia / all subheadings

12. or/ #9-#11

13. exp Unguentum / all subheadings

14. exp Plaster / all subheadings

15. exp Emplastrums/ all subheadings

16. exp Emplastrums therapy/ all subheadings

17. patch(贴膏) in all Fields

18. patch(贴剂) in all Fields

19. patch(膏贴) in all Fields

20. patch(巴布膏) in all Fields

21. or/ #13-#20

22. Human

23. #7 and #12 and #21 and #22

**Appendix II**

**Sources of 80 kinds of traditional Chinese herbal patches.**

[1] X. Wang, Y. Cao, J. Pang, J. Du, C. Guo, T. Liu, S. Wei, Y. Zheng, R. Chen, and H. Zhan, “Traditional chinese herbal patch for short-term management of knee osteoarthritis: a randomized, double-blind, placebo-controlled trial,” *Evidence-Based Complementary and Alternative Medicine*, vol.2012, article ID 171706, 2012.

[2] H. Yin, Y. Ma, and J.W. Wang, “Patching therapy for the treatment of knee osteoarthritis 60 Cases,” *Forum on Traditional Chinese Medicine*, vol.26, no.5, pp. 17-18, 2011.

[3] Z. Wang, Z.C. Xia, W.M. Yuan, G.H. Pi, R. Sun, and X.C.Wang, “Clinical observation of Chuan Zhi patch for the treatment of lumbar spine bone hyperplasia,” *Chinese Journal of Aesthetic Medicine*, vol.20, no.2, pp. 22-23, 2011.

[4] Y. Zhang, Y.L. Xie, Q.T. Zhang, T. Ma, and L.J. Li, “Clinical observation of Qu Yu Zhi Tong patch for the treatment of osteoarthritis of the knee,” *Inner Mongolia in Traditional Chinese Medicine*, vol.30, no.14, pp. 29-30, 2011.

[5] J. Shen, J.Y. Chen, X.L. Yun, Q.Q. Zhu, and Y.X. Li, “Clinical comparative study on self-made Tongluo Qing Bi patch for the treatment of knee osteoarthritis,” *Journal of Guiyang College of Traditional Chinese Medicine*, vol.33, no.2, pp. 37-38, 2011.

[6] Y. Cao, H. Zhan, J. Pang, F. Li, S. Xu, J. Gao, Z. Xu, G. Li, T. Liu, C. Guo *et al.*, “Individually integrated traditional Chinese medicine approach in the management of knee osteoarthritis: study protocol for a randomized controlled trial, *Trials*, vol.12, no.1, pp.160, 2011.

[7] G. Liu, “Efficacy observation of Qizheng Xiaotong patch for the treatment of osteoarthritis of the knee,” Contemporary Medicine, vol.17, no.21, pp. 160-161, 2011.

[8] C.H. Zeng, X.H. Su, and J.L. Zhao, “Tong Yu patch for the treatment of osteoarthritis of the knee 25 cases,” *Hunan* *Journal of Traditional Chinese Medicine*, vol.26, no.4, pp. 51-53, 2010.

[9] Y. Zhang, and Y.M. Xin, “Clinical observation of homemade Wentong patch for the topical treatment of osteoarthritis of the knee,” *Chinese Manipulation ＆ Rehabilitation Medicine*, vol.1, no.16, pp. 52-53, 2010.

[10] H.Y. Yao, G.X. Cai, and B.Y. Liu, “Chao Wei Zhong Tong Xiao patch for the treatment of osteoarthritisof the knee 30 cases,” *Hunan Journal of Traditional Chinese Medicine*, vol.24, no.4, pp. 40-41, 2010.

[11] L. Zhang, L. Dong, D.Z. Zhao, and D.J. Zhang, “Observation on treating osteoarthritis pain with Gu Ci Xiao cataplasm,” *Chinese Journal of the Practical Chinese with modern Medicine*, vol.23, no.7, pp. 12-13,2010.

[12] Z. Y. Guan, “Zhuanggu Tongbi cream in treatment of knee osteoarthritis clinical research,” *Clinical Journal of Chinese Medicine*, vol.2, no.7, pp. 14-15+17, 2010.

[13] J. Liao, F. M. Lv, and Q. C. Meng, “Clinical efficacy of Re Yun I recipe to treat type of Yang Xu Han Nin in knee osteoarthritis,” *Xinjiang Medical University*, vol.33, no.8, pp. 988-989+992, 2010.

[14] J. Qi, and J.H. Yu, “Report of 100 cases of Gu Shang Xiao Yan Zhen Tong patch for the treatment of patellar pain,” *The Journal of Traditional Chinese Orthopaedics and Traumatology*, vol.22, no.7, pp. 50+19, 2010.

[15] Z.Q. Liang, Q.F. Meng, R. Fang, Y.C. Song, and Y.J. Deng, “Shang Ke Hei Yao patch for the treatment of knee osteoarthritis 46 cases,” *Henan Traditional Chinese Medicine*, vol.30, no.1, pp. 53-54, 2010.

[16] Z.A. Tan, and J.W. Li, “Clinical study of Jinghuang for knee plaster on external application for knee osteoarthritis,” *Journal of TCM Univ. of Hunan*, vol.30, no.7, pp. 73, 2010.

[17] L. C. Su, “Clinical observation of Jiegu patch for the treatment of osteoarthritis of the knee effusion,” *Chinese Medicine Morden Distance Education of China*, vol.8, no.17, pp. 246-247, 2010.

[18] W.J. Yu, Y.F. Cao, G.Y. Zhang, and L.G. Feng, “Clinlical observation on the curative effect of blistering theray for the treatment knee osteoarthritis,” *The Journal of Traditional Chinese Orthopaedics and Traumatology*, vol.22, no.6, pp. 8+11, 2010.

[19] P.Wang, E.P. Gu, and H.Y. Cao, “Clinical observation of Huo Xue Hua Yu patch for the treatment of early and mid-osteoarthritis of the knee,” *Jilin Traditional Chinese Medicine*, vol.30, no.6, pp. 491-493, 2010.

[20] X.H. Du, “Clinical observation of Gu Bi Xi Tong Papua patch for the topical treatment of knee osteoarthritis,”*Journal of New Chinese Medicine*, vol.42, no.9, pp. 55, 2010.

[21] J.J. Kuang, “Zhong Tong Xiao Papua patch for the treatment of osteoarthritis of the knee 48 cases,” *Hunan Journal of Traditional Chinese Medicine*, vol.24, no.3, pp. 62-63, 2010.

[22] L.J.Chen, S.M. Wang, and Y. Wang, “Famous and old herbalist of Chen Zhankui's Zhi Tong Hua Yu patch for the treatment of bone and joint diseases 83 cases,” *Chinese Medicine Morden Distance Education of China*, vol.8, no.8, pp. 12-13, 2010.

[23] X.L. Sun, “Huangbo Wuwei patch for the treatment of knee osteoarthritis 90 cases,” *Modern Traditional Chinese Medicine*, vol.29, no.5, pp. 46-47, 2009.

[24] Y.F. Cheng, S.F. Gu, and M. Liu, “San Huang patch for the topical treatment of knee osteoarthritis 63 cases, *Shenzhen Journal of Integrated Traditional and Western Medicine*, vol.19, no.3, pp. 190+200, 2009.

[25] M.T. Yan, and Y. Yan, “Chinese herbal patch for the topical treatment of bone hyperplasia 75 cases,” *Journal of External Therapy of TCM*, vol.18, no.3, pp. 26-27, 2009.

[26] C.Q. Zhou, S.W. Liu and K.L. Liu, “Traditional Hei Yao patch for the treatment of bone proliferative diseases 120 cases,” *Shanxi Journal of Traditional Chinese Medicine*, vol.30, no.4, pp. 433-434, 2009.

[27] H.M. Li, F.L. Liu, and S.Q. Guo, “Yao Tong Ning patch for the topical treatment of osteoarthritis of the knee 48 cases,” *Journal of External Therapy of TCM*, vol.18, no.6, pp. 28, 2009.

[28] M.X. Zhang, and G.H. Fang, “Clinical analysis of Tihe Gu Tong Tie patch for the treatment of lumbar vertebrae osteoarthritis,” *Modern Hospital*, vol.9, no.10, pp. 36-37, 2009.

[29] M. Li, P.F. Xiu, and C.G. Zhu, “Ba Wei patch for the treatment of knee osteoarthritis 80 cases,” *Shanxi Journal of Traditional Chinese Medicine* vol.30, no.4, pp. 433-434, 2009.

[30] L.P. Yin, Q.Z. Zhou, J.G. Wu, X.M. Cheng, and Y.J. Gu, “Clinical observation of TCM topical treatment of proliferative knee osteoarthritis 30 cases by method of promoting blood circulation and eliminating phlegm,” *Henan Traditional Chinese Medicine*, vol.29, no.9, pp. 887-888, 2009.

[31] S.G. Liu, and Y.G. Jiang, “Hei Hu patch for the treatment of knee osteoarthritis 260 cases,” *Shanxi Journal of Traditional Chinese Medicine*, vol.319, no.7, pp. 820-821, 2008.

[32] G.S. Ding, Q.R. Shen, and H.Y. Xie, “Preparation and clinical observation of Kang Wei Tong Bi patch,” *Chinese Anchive of Traditional Chinese Medicine*, vol.26, no.3, pp. 582-583, 2008.

[33] J. F. Guo, Z. Q. Du，T. Z. Bao, X. Z. Qiu, X.M. Xiu, Y.W. Luo, Q.R. He, and J.H. Zhang, “Clinical study of Xiong Zhi Tong Yu Xiao San Tie on knee pain(Blood stasis syndrome) induced by osteoarthritis,” *World Journal of Integrated Traditional and Western Medicine*, vol.26, no.8, pp. 468-470, 2008.

[34] H. Wen, W.H. Zhao, X.C. Li, et al, “Clinical observation of Xi Tong Gao for the treatment of osteoarthritis of the knee,” *Jilin Journal of Traditional Chinese Medicine*, no.9, pp. 664, 2008.

[35] J. G. Zhang, D.M. Fu, and C.L. Yang, “The clinical research about the therapy of bone arthritis of knee joint by Zhitong Tougu Ointment,” *China Medical Heral*, vol.8, no.5, pp. 45-46, 2008.

[36] Y.G. Wang, D.Y. Wei, F.C. Wang, and X.L. Liang, “Feng Shi Gu Tong patch for the treatment of osteoarthritis,” *China's Naturopathy*, vol.16, no.2, pp. 16, 2008.

[37] M. C. Pan, “Gu Tong Ning patch for the treatment of knee osteoarthritis 216 cases,” *China Foreign Medical Ttreatment*, vol.27, no.24, pp. 15-16, 2008.

[38] Z. Dong, “Clinical research of Shujin patch for the treatment of osteoarthritis of the knee,” *Journal of Fujian College of Traditional Chinese Medicine*, vol.17, no.3, pp. 46-47, 2007.

[39] A. L. Zhao, “Report of 112 cases about Gu Bi Tong patch for the topical treatment of bone and joint disease,” *Zhejiang University of Traditional Chinese Medicine*, vol.177, no.1, pp. 94-95, 2007.

[40] J. P. Chen, “Chinese herbal patch for the topical treatment of osteoarthritis of the knee 90 cases,” *Journal of External Therapy of TCM*, vol.16, no.6, pp.11, 2007.

[41] L. Lin, “Clinical observation of Shang Ke Xiao Yan patch for the topical treatment of the cold-damp stasis of knee osteoarthritis,” *Morden Medicine drug and Health*, vol.22, no.18, pp. 2786-2787, 2006.

[42] C. Q. Long, “Clinical observation of Guan Jie Yan patch for the topical treatment of knee osteoarthritis,” *Modern Journal of Integrated Traditional Chinese and Western Medicine*, vol.15, no.18, pp. 2494, 2006.

[43] Y. X. Zheng, H. S. Zhan, H. Zhang, S.G. Niu and Z.J. Zhuang, “Qi-zheng Qing-peng slurry for treatment of the knee osteoarthritis: A randomized,controlled clinical research,” *China Journal of Orthopaedics and Traumatology*, vol.19, no.5, pp. 316-317, 2006.

[44] B. Shi, and G. Li, “Clinlical study on Fu Fang Huo Xue patch for the topical treatment of osteoarthritis of the knee (0 - II period),” *Journal of External Therapy of TCM*, no.5, pp. 3-5,2006.

[45] K. H. Feng, “Clinical efficacy judgment of Gu Ci patch for the treatment of osteoarthritis of the knee,” *Health Vocational Education*, vol.09, pp. 141-142, 2006.

[46] M. P. Li, Q.B. Han, Q. Q. Wang, Y.F. Zhang, and Q.S. Ma, “Homemade Mei Po Zheng Gu patch for the treatment of bone hyperplasia 132 cases,” *Clinical Medicine of Traditional Chinese Medicine*, vol.17, no.2, pp. 181, 2005.

[47] J.X. Wu, C. X. Huang, J.Y. Lin, and Z. L. Tang, “Clinical observation of Xi Tong Ning patch for the topical treatment of knee osteoarthritis,” *China Journal of Orthopaedics and Traumatology*, vol.18, no.5, pp. 314, 2005.

[48] F. Li, and J. M. Wang, “Tong Ying patch for the treatment of osteoarthritis of the knee 120 Cases,” *Journal of External Therapy of TCM*, no.6, pp. 26-27, 2005.

[49] J. M. Wang, and L. W. Wu, “Zheng Tong Xiao Yan patch for the treatment of osteoarthritis 66 cases,” *Heilongjiang Journal of Traditional Chinese Medicine*, no.6, pp. 13-14, 2005.

[50] Q. Zhang, J. Xiao, and L. Dun, “Clinical study of Shexiang tongbi pupua patch for the treatment of the symdrone of liver and kedney defficious and stens-muslcuar stasis of oateoarthritis,” *Chinese Journal of Clinical Pharmacology and Therapeutics*, vol.18, no.8, pp. 953-956, 2005.

[51] Z. J. Dou, J.F. Wang, and H.Guo, Preparation and clinical observation of Gu Ci Xiao patch Ⅰ,” *Clinical Journal of Traditional Chinese Medicine Medicine*, vol.17, no.3, pp. 228-229, 2005.

[52] H. J. Tao, “Young's Xiao Zhong Zhi Tong patch for the treatment of bone hyperplasia of lumbar spine 200 cases,” *Jangsu Journal of Traditional Chinese Medicine*, vol.26, no.3, pp. 39, 2005.

[53] J. P. Liu, M.H. Yang, Y. Qiu, and T.J. Huang, “Observed clinical efficacy of traditional Chinese medicine for the topical treatment of osteoarthritis of the knee,” *Xinjiang Traditional Chinese Medicine*, vol.22, no.1, pp. 15-16, 2004.

[54] F. X. Zhang, “Bi Tong patch for the treatment of bone hyperplasia,” *Inner Mongolia Journal of Traditional Chinese Medicine*, no.3, pp.3, 2003.

[55] X. S. Zhou, and D.B. Yi, “Pharmacodynamical research on anti-inflammatory of Wei Ling Xian,” *Chinese Journal of Clinical Medicine and Pharmacy(Beijing)*, vol.4, pp. 12-13, 2003.

[56] C. Wang, “Clinical research of Fu Fang Yan Ning patch for the treatment of bone hyperplasia,” *Chinese Journal Traditional Medicine Traumatology & Orthopedics*, no.8, pp. 46-48, 2002.

[57] S. C. Bai, Z.G. Wang, F.J. Zhang, H. Li, and L.H. Zeng, “Clinical research of Gu Tong patch in curing proliferative arthritis of knee joint,” *Chinese Journal of Traditional Medicine Traumatology & Orthopedics*, no.8, pp. 474-475, 2002.

[58] E. P. Xu, G.L. Li, Q.H. Yang, and S.Q. Jang, “Gu Bi patch for the treatment of knee osteoarthritis 78 cases,” *New Traditional Chinese Medicine*, vol.34, no.10, pp. 56, 2002.

[59] Y. Cao, J.P. Yao, J.N. Dong, Y.H. Lin. and J.Z. Gao, “Clinical observation of Qu Tong patch for the treatment of degenerative knee joint disease,” *Liaoning Journal of Traditional Chinese Medicine*, vol.29, no.8, pp. 474-475, 2002

[60] Q. H. Zhou, “Summury of Tong Bi Zhi Tong patch for the treatment of primary osteoarthritis 120 cases,” *Hunan Journal of Traditional Chinese Medicine*, vol.18, no.1, pp. 13-14, 2002.

[61] J. Liu, “Homemade Gu Bi patch for the treatment of bone hyperplasia 62 cases,” *Journal of External Therapy of TCM*, no.4, pp. 23, 2001.

[62] S. X. Sun, W. Hou, and J.S. Liu, “Gu Ci patch for the treatment of knee arthropathy 60 cases,” *Liaoning Journal of Traditional Chinese Medicine*, vol.28, no.1, pp.30, 2001.

[63] S. L. Yang, and C.J. Zhou, “Gu Ci Xiao Tong patch for the treatment of bone hyperplasia 637 cases,” *China Journal of Orthopaedics and Traumatology*, no.7, pp. 48, 2001.

[64] W. D. Gong, “Wen Jin Tong Luo patch for the topical treatment of hypertrophic arthritis of the knee 51 cases,”*Jangsu Journal of Traditional Chinese Medicine*, no.5, pp. 29, 2001.

[65] Z. Tang, “Tongbi Huoxue patch for the treatment of ostoearthritis of the limb 96 cases,” *Information on Traditional Chinese Medicine*, no.3, pp. 41, 2001.

[66] F.Q. Liu, “Ling Gu Xue patch for the treatment of osteoarthralgia 278 Cases,” *Journ al of External Therapy of TCM*, no.2, pp. 35, 2001.

[67] J.L. Yan, F.R. Cheng, X.J. Wang, J.Y. Tang, B.G. Wu and Y.X. Ling, “Clinlical observation of Le E acupoint patch for the treatment of knee osteoarthritis 50 cases,” *Chinese Journal of Traditional Medical Science and Technology*, no.6, pp. 407-409, 2000.

[68] W.Q. Li, S.L.Wang, and B.T. Zhu, “Clinical observation of Gu Shang Feng Shi patch for the treatment of knee osteoarthrosis,” *Chinese Journal Traditional Medicine Traumatology & Orthopedics*, vol.10, no.4, pp. 44-45, 2000.

[69] X. H. Zhou, D. Lin, W.C. Meng, F. Li, and H.S. Lin, “Clinical study of Gu Ci Ning patch for the treatment of deenerative joint disease 100 cases,” *Guangzhou University of Traditional Chinese Medicine*, vol.17, no.1, pp. 30-34, 2000.

[70] B, Xu, and J.F. Lu, “Clinical research of Fu Fang San Sheng patch for the treatment of knee ostoarthritis,” Clinical Medicine, vol.20, no.9, pp. 13-14, 2000.

[71] H.S.Tian, and J.Q.Lu, “Shujin Xiaotong Wanying patch for the treatment of bone hyperplasia of cervical and lumbar spine 285 cases,” Guang Ming Zhong Yi, vol.15, no.5, pp. 51-52, 2000.

[72] Y. L. Yin, X.Q. Wang, and Q.H. Li, “Ru Gui patch for the topical treatment of knee osteoarthritis 100 cases,” *Journal of Henan college of Traditional Chinese Medicine*, no.3, pp. 38,1999.

[73] Y. Q. Yang, “Clinical study of Gu Zeng Sheng Zheng Tong for the treatment of osteoarthritis,” *Traditional Chinese Drug Research & Clinical Pharmacology*, vol.10, no.3, pp. 138-140, 1999.

[74] F. T. Hao, Q.L. Hao, Z.G. Jiang, G.W. Xiu, C.S. Yan, and T. Yan, “Effeciacy observation of Feng Shi Shang Tong patch for the treatment of osteoarthritis of the knee,” *Journal of External Therapy of TCM*, vol.8, no.6, pp. 43-44, 1999.

[75] F. T. Hao, Z.G. Jiang, C.Y. Tian, G.W. Xiu, C.S. Yan, T. Yan, and G.P. Huang, “Effeciacy observation of Fufang Lingzhi patch for the treatment of osteoarthritis of the knee,” *Research of Traditional Chinese Medicine*, vol.15, no.6, pp. 17-19, 1999.

[76] X.P. Ren, “Gu Ci Ting patch for the treatment of bone hyperplasia in 528 patients,” *Shanxi Journal of Traditional Chinese Medicine*, vol.14, no.4, pp. 41, 1998.

[77] H. J. Wang, and X.Z. Fan, “Clinical observation Gu Zhi Zhi Tong patch for the treatment of bi-arthralgia of cervical spondylos,” *Practical Traditional Chinese Medicine*, vol.11, no.12, pp. 1127, 1998.

[78] X.L. Du, and H. Zhang, “Clinical research of Ji Li Huo Xue patch for the treatment of knee osteoarthritis,” *Journal of Shandong University of Traditional Chinese Medicine*, vol.21, no.6, pp. 443-445, 1997.

[79] L. Chen, Z.E. Quan, F. Zhao, W.X.Wang and Z.Q. Huo, “Jing Zhui patch for the topical treatment of cervical spondylosis 80 cases,” *Hunan Journal of Traditional Chinese Medicine*, vol.12, no.4, pp. 22-23, 1996.

[80] W. S. Lian, and W.Q. Pan, “Wu Long Wei Ling patch for the treatment of bone hyperplasia 1250 cases,” *Shandong Journal of Traditional Chinese Medicine*, vol.14, no.9, pp. 406-407, 1995.

**6 kinds of patches in 2010 version of China Pharmacopoeia(One)**

She Xiang Zheng Tong patch

Die Da Zheng Tong patch

Shang Shi Zhi Tong patch

Shao Lin Feng Shi Die Da patch

Gou Pi patch

Hong Yao patch

**Appendix Ⅲ Methodological assessment about each risk of bias of TCHPs for OA in included RCTs and CCTs**

| First author (year) | Sequence generation | Allocation concealment | Blinding | Incomplete outcome data | Selective reporting | Other bias |
| --- | --- | --- | --- | --- | --- | --- |
| Bai SC 2004 | ? | ? | ? | ? | ? | ? |
| Cao Y 2002 | ? | ? | ? | ? | ? | ? |
| Chen JP 2007* | ? | ? | ? | ? | ? | − |
| Cheng FY 2009* | ? | ? | ? | ? | ? | ? |
| Ding GS 2008 | ? | ? | ? | ? | ? | ? |
| Dong Z 2007* | ? | ? | ? | ? | ? | ? |
| Dou JZ 2005 | ? | ? | ? | ? | ? | ? |
| Du XL 1997 | ? | ? | ? | ? | ? | ? |
| Gou JF 2008 | + | + | ? | ? | ? | + |
| Guan ZY 2010 | + | ? | ? | ? | ? | ? |
| Kuang JJ 2010# | - | ? | ? | ? | ? | ? |
| Li M 2009 | ? | ? | ? | ? | ? | ? |
| Liang ZQ 2010 | ? | ? | ? | ? | ? | ? |
| Liao J 2010 | ? | ? | ? | ? | ? | ? |
| Lin L 2006 | + | + | ? | − | ? | ? |
| Liu G 2011 | ? | ? | ? | ? | ? | ? |
| Liu JP 2004 | ? | ? | ? | − | ? | ? |
| Liu SG 2008* | ? | ? | ? | ? | ? | ? |
| Long ZQ 2006 | ? | ? | ? | ? | ? | ? |
| Ma KH 2006* | ? | ? | ? | ? | ? | − |
| Shen J 2011 | ? | ? | ? | ? | ? | ? |
| Shi B 2006 | ? | ? | ? | ? | ? | ? |
| Su LC 2010 | ? | ? | ? | ? | ? | ? |
| Su XL 2009 | ? | ? | ? | ? | ? | ? |
| Tan ZA 2010 | ? | ? | ? | ? | ? | ? |
| Tang Z 2001 | ? | ? | ? | ? | ? | ? |
| Wang C 2002 | ? | ? | ? | ? | ? | ? |
| Wang GY 2008 | ? | ? | ? | ? | ? | ? |
| Wang HJ 1998 | ? | ? | ? | ? | ? | ? |
| Wang JW 2005* | ? | ? | ? | ? | ? | − |
| Wang P 2010 | + | ? | ? | ? | ? | ? |
| Wang XZ 2012 | + | + | + | + | − | + |
| Wang YY 2006 | ? | ? | ? | − | ? | ? |
| Wang Z 2011 | ? | ? | ? | ? | ? | ? |
| Wen H 2008* | ? | ? | ? | ? | ? | ? |
| Xu B 2000* | ? | ? | ? | ? | ? | − |
| Xu EP 2002 | ? | ? | ? | ? | ? | ? |
| Yan JL 2000 | ? | ? | ? | ? | ? | ? |
| Yang YQ 1999 | + | ? | ? | ? | ? | ? |
| Yao TH 2008 | ? | ? | ? | ? | ? | ? |
| Yin H 2011 | ? | ? | ? | ? | ? | ? |
| Yin LP 2009 | ? | ? | ? | ? | ? | ? |
| Yin YL 1999 | ? | ? | ? | ? | ? | ? |
| Yu WJ 2010 | ? | ? | ? | ? | ? | ? |
| Zeng CH 2010 | ? | ? | ? | ? | ? | ? |
| Zhang JG 2008 | − | ? | ? | ? | ? | ? |
| Zhang L 2010# | ? | ? | ? | ? | ? | ? |
| Zhang MX 2009 | ? | ? | ? | ? | ? | ? |
| Zhang Q 2005 | + | + | + | − | ? | + |
| Zhang Y 2007 | ? | ? | ? | + | ? | ? |
| Zhang Y 2010* | ? | ? | ? | ? | ? | ? |
| Zhang Y 2011 | ? | ? | ? | ? | ? | ? |
| Zhao AL 2007* | ? | ? | ? | ? | ? | − |
| Zhen YX 2006 | + | + | ? | ? | ? | ? |
| Zhou QH 2002 | ? | ? | ? | ? | ? | ? |
| Zhou XH 2000 | + | ? | ? | ? | ? | ? |

Note: *, non-randomized controlled trials; # , interrupted-time-series study; +, low risk; ?, unclear risk; −, high risk.
